# Unanticipated domain requirements for *Drosophila* Wnk kinase *in vivo*

**DOI:** 10.1371/journal.pgen.1010975

**Published:** 2023-10-11

**Authors:** Prathibha Yarikipati, Sima Jonusaite, John M. Pleinis, Carihann Dominicci Cotto, David Sanchez-Hernandez, Daryl E. Morrison, Suhani Goyal, Jeffrey Schellinger, Clothilde Pénalva, Jennifer Curtiss, Aylin R. Rodan, Andreas Jenny

**Affiliations:** 1 Department of Developmental and Molecular Biology, Albert Einstein College of Medicine, New York, United States of America; 2 Molecular Medicine Program, University of Utah, Salt Lake City, Utah, United States of America; 3 Department of Internal Medicine, Division of Nephrology, University of Texas Southwestern, Dallas, Texas, United States of America; 4 Department of Cell & Developmental Biology, New Mexico State University, Las Cruces, New Mexico, United States of America; 5 Department of Internal Medicine, Division of Nephrology and Hypertension, University of Utah, Salt Lake City, Utah, United States of America; 6 Department of Human Genetics, University of Utah, Salt Lake City, Utah, United States of America; 7 Medical Service, Veterans Affairs Salt Lake City Health Care System, Salt Lake City, Utah, United States of America; 8 Department of Genetics, Albert Einstein College of Medicine, New York, New York, United States of America; Mayo Clinic, UNITED STATES

## Abstract

WNK (With no Lysine [K]) kinases have critical roles in the maintenance of ion homeostasis and the regulation of cell volume. Their overactivation leads to pseudohypoaldosteronism type II (Gordon syndrome) characterized by hyperkalemia and high blood pressure. More recently, WNK family members have been shown to be required for the development of the nervous system in mice, zebrafish, and flies, and the cardiovascular system of mice and fish. Furthermore, human WNK2 and *Drosophila* Wnk modulate canonical Wnt signaling. In addition to a well-conserved kinase domain, animal WNKs have a large, poorly conserved C-terminal domain whose function has been largely mysterious. In most but not all cases, WNKs bind and activate downstream kinases OSR1/SPAK, which in turn regulate the activity of various ion transporters and channels. Here, we show that *Drosophila* Wnk regulates Wnt signaling and cell size during the development of the wing in a manner dependent on Fray, the fly homolog of OSR1/SPAK. We show that the only canonical RF(X)V/I motif of Wnk, thought to be essential for WNK interactions with OSR1/SPAK, is required to interact with Fray *in vitro*. However, this motif is unexpectedly dispensable for Fray-dependent Wnk functions *in vivo* during fly development and fluid secretion in the Malpighian (renal) tubules. In contrast, a structure function analysis of Wnk revealed that the less-conserved C-terminus of Wnk, that recently has been shown to promote phase transitions in cell culture, is required for viability *in vivo*. Our data thus provide novel insights into unexpected *in vivo* roles of specific WNK domains.

## Author summary

Members of the conserved WNK kinase family are large proteins that have essential developmental roles, as well as crucial functions in the regulation of ion transporters in kidneys via the intermediary kinases OSR1 and SPAK. Using *Drosophila* as a model system, we show that the fly homolog of the intermediary kinases, Fray, acts downstream of Wnk during the development of the fly wing to regulate cell size. Surprisingly though, our work reveals that Wnk does not need the canonical motif that binds to Fray for function *in vivo*. In contrast, we find an essential function for the less-well conserved C-terminus of Wnk that recently was shown to form membraneless organelles. Our findings thus reveal unanticipated roles of WNK domains.

## Introduction

Members of the WNK (With no Lysine [K] kinase) family of protein kinases are characterized by the atypical placement of the catalytic lysin (K) in subdomain I of their kinase domain rather than subdomain II as in conventional kinases [1–3]. WNK kinases control ion reabsorbtion in the kidney and are also known for their role in the regulation of cellular volume [4–7]. In humans, dominant mutations in WNK1 and WNK4, two of the four mammalian paralogs, cause autosomal dominant Gordon’s syndrome (a.k.a. Pseudohypoaldosteronism Type II or familial hyperkalemic hypertension). Gordon syndrome is characterized by hypertension and hyperkalemia, reflecting the fact that WNK kinases are critical regulators of Na^+^/K^+^/Cl^-^ co-transporters (N(K)CCs) controlling ion reabsorbtion [8–11]. WNKs regulate N(K)CCs and the related potassium chloride cotransporters (KCCs) by activating the functionally redundant, intermediary kinases SPAK (Ste20/SPS1-related proline/alanine-rich kinase) and OSR1 (Oxidative stress responsive-1), which in turn activate sodium-coupled chloride transporters (NCCs) and sodium-potassium-2-chloride cotransporters (NKCCs), while inhibiting KCCs [12–16]. WNK function is thus important for the control of cell volume, transepithelial ion flux, and the regulation of intracellular Cl^-^ concentration. Importantly, the WNK-OSR1/SPAK-N(K)CC/KCC axis is highly conserved from *C*. *elegans*, *Drosophila*, and Zebrafish to mammals (17, 18). In particular, in principal cells of the Malpighian tubules, the *Drosophila* renal epithelium, Wnk acts through Frayed (Fray; the homolog of OSR1/SPAK) and an NKCC, Ncc69, to regulate ion flux and fluid secretion from the blood into the lumen of the tubule to produce urine [17–20]. Functional and structural studies have shown that OSR1 and SPAK interact via their conserved C-terminal (CCT) domains with short RF(x)V/I motifs present in upstream WNK kinases as well as the downstream ion transporters (reviewed in [21,22]).

Wnk kinases also have important functions during development. *Wnk1* knock-out mice die around day E9.5/10.5 with severe angiogenesis and cardiac developmental defects [23]. Zebrafish knock-down studies of *Wnk1* also have revealed functions of Wnks during angiogenesis and neural development [24,25]. In humans, mutations in the HSN2 exon of WNK1 have been linked to Hereditary Sensory and Autonomic Neuropathy type II (HSANII), characterized by early onset neuropathy and a reduction of myelinated nerve fibers, amongst other defects [26–28]. Consistently, knockdown of the *hsn2* isoform of *wnk1b* in Zebrafish severely affected the development of the neuromasts of the lateral line organ, likely via upregulation of *kcc2* [29]. During *Drosophila* development, Wnk and Fray are required for neurogenesis in the embryo, and the formation of the adult cuticle [30], in a manner dependent on Fray. Specifically, their activity induces the LIM-homeobox transcription factor Arrowhead, the homolog of vertebrate LHX8, specifying the primordia for adult abdominal structures [30]. Furthermore, we previously found that Wnk modulates canonical Wnt/β-Catenin signaling upstream of the adapter protein Dishevelled during wing development. However, epistasis with Fray has not been addressed in that case [31,32]. Importantly, the function of Wnk in Wnt signaling and neural development via LHX8 is conserved in mammalian cell culture [30–33]. Most recently, Wnk and Fray also have been shown to regulate the circadian rhythm in adult flies (34, 35).

The WNK kinase domain is highly conserved between paralogs and across species, whereas the large portion distal to it, which is not required for kinase activity [1,36,37], is poorly conserved. For example, human WNKs 1–4 share 83–91% sequency identity within the kinase domain, and the *Drosophila* Wnk kinase domain is 73% identical to human WNK1 [18]. In contrast, the human WNK1 kinase distal area is only 24% identical to the human WNK3, and is 22% identical to the corresponding region in Drosophila [38]. Common features of the distal part in both mammalian and *Drosophila* WNKs are high disorder tendency and the presence of multiple coiled-coil domains. Interestingly, recent work by Boyd-Shiwarski [38] implicated this part of WNK1, including its coiled-coil domain, in phase separation into membraneless organelles in response to hypertonic stress and cell volume regulation in cultured cells. *Drosophila* WNK also phase separates in response to hypertonic stress, which requires the distal region of the protein [38].

Here, we show that Wnk acts upstream of Fray also to modulate Wnt signaling and cell size during wing development. Although most functions of Wnk in flies are mediated by Fray, and the single canonical RF(x)V/I motif of Wnk is required for its interaction with Fray *in vitro*, the motif unexpectedly is dispensable *in vivo* for development, viability and fluid secretion in the Malpighian tubules. Our data thus suggest that additional factors stabilize the Wnk-Fray signaling module *in vivo*. Intriguingly, detailed structure function studies show that, in contrast to two coiled-coil regions and a large central portion of Wnk, its disordered C-terminus is essential for Wnk function *in vivo*.

## Results

### Fray acts downstream of Wnk in the fly wing

**Fig 1:**
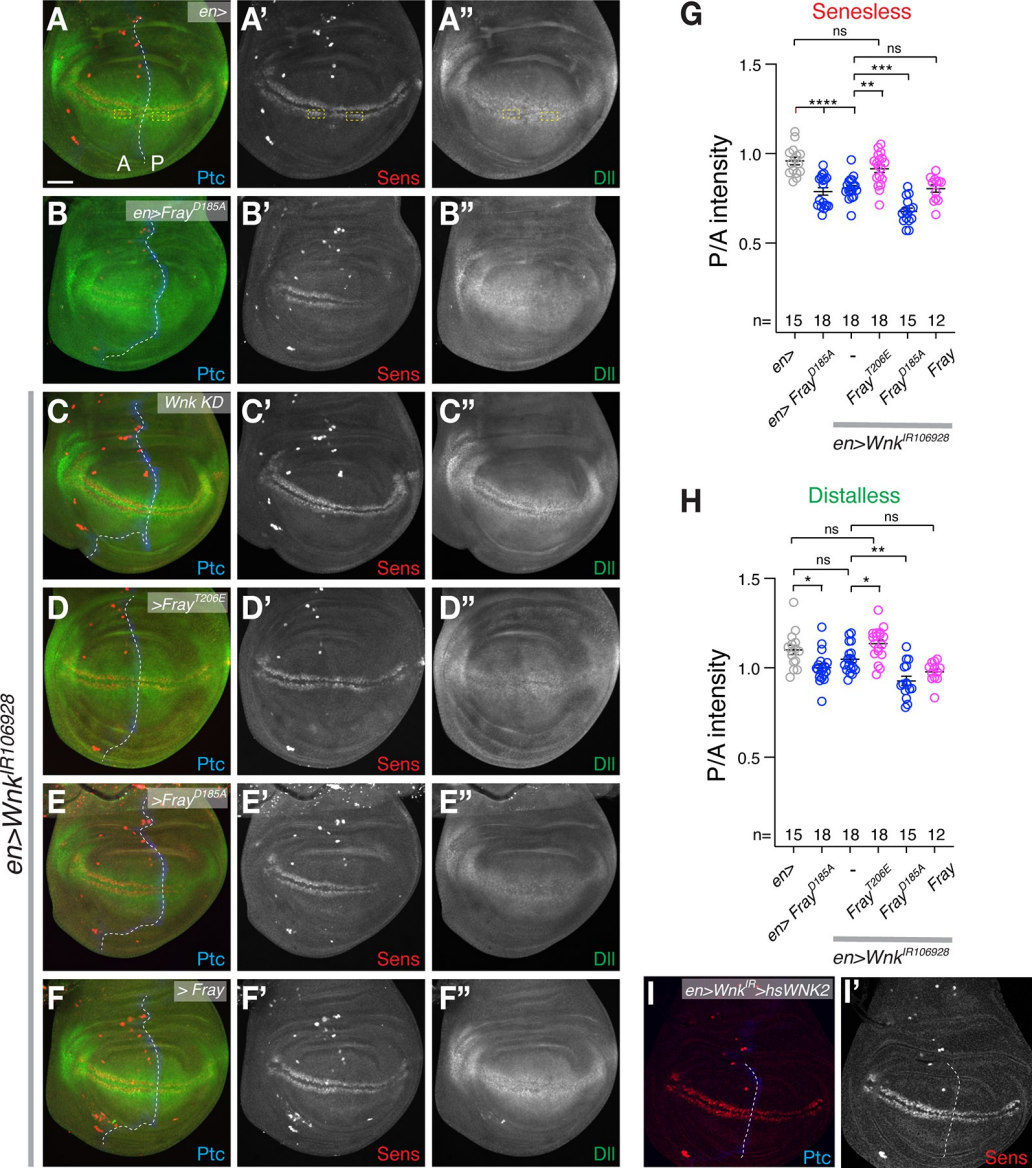
Fray acts downstream of Wnk to modulate Wnt signaling. (**A**) In control wings (*en-Gal4* [*en>*]), Sens (red; A’) is expressed in two narrow stripes abutting the Wg expression domain on either side of the dorsoventral compartment boundary, while Dll (green; A”) shows a broader expression pattern in the wing blade primordium. The anterior (A)—posterior (P) compartment boundary marked by Patched (Ptc; blue) is outlined by a dotted white line. (**B**) Expression of dominant negative *Fray*^*D185A*^ specifically on the posterior side reduces Sens (red) and Dll (green) expression. (**C**) Knockdown of *Wnk* in the posterior compartment (*en>Wnk*^*IR106928*^ [Wnk KD]) reduces levels of Sens (C’), while Dll shows a tendency towards lower expression (C”). **(D)** The reduction of Sens and Dll by Wnk knockdown is suppressed by expression of constitutively active *Fray*^*T206E*^. (**E**) Expression of dominant negative *Fray*^*D185A*^ enhances the effect of *Wnk* knockdown. **(F)** WT-Fray is not sufficient to suppress the effect of *Wnk* knockdown. **(G, H)** Quantification of posterior to anterior signal intensity of Sens (G) and Dll (H). Plotted are ratios of posterior to anterior signal corresponding to the areas outlined by dotted yellow boxes in A (see also methods). One-way ANOVAs (Tukey correction) P <0.0001. ****, P <0.0001; ***, P <0.001; **, P <0.01; *, P <0.05; ns, not significant. Only key significances are indicated. **(I)** Human *WNK2* rescues the reduction of Sens by *Wnk* knockdown. For all images, greyscale images show indicated single channels. Scale bar: 50μm.

The best understood roles of WNKs are mediated by OSR1/SPAK/Fray Ste20 kinases. We thus asked if Wnk’s role in Wnt signaling is mediated by Fray. The loss of Wnk during development of the *Drosophila* wing causes wing margin defects due to a reduction in Wnt signaling manifest by reduced expression of the transcription factor Senseless (Sens), a high threshold, direct target of the fly Wnt Wingless (Wg) [31]. Sens normally is expressed in two narrow stripes in the wing primordium where it is required to specify margin structures including bristles (Fig 1A and 1A’). The low threshold Wg target Distalless (Dll) is expressed in a wider area of the wing primordium (Fig 1A and 1A”) [39]. The GAL4-UAS system allows gene knockdown and expression in specific cells, with the yeast GAL4 transcription factor expressed in a specific cell type driving expression of a transgene or dsRNA downstream of UAS-Gal4 binding sites [40]. Expression of dominant negative, kinase dead Fray^D185A^ in the posterior compartment using *enGal4* reduces Sens and Dll expression (Fig 1B–1B”); quantification of posterior to anterior expression ratios of Sens and Dll in G, H, respectively; see S1 Table for exact genotypes). Similarly, knockdown of *Wnk* in the posterior compartment using *en-Gal4* reduces Sens expression (*Wnk KD* in Fig 1C–1C’; G for quantification) with Dll expression showing a tendency towards lower levels too (Fig 1C–1C”; H for quantification). To assess if Wnk acts though Fray in the wing, we tested whether overexpression of Fray can suppress the effect of knockdown of Wnk. Indeed, coexpression of constitutively active *UAS-Fray*^*T206E*^, but not WT-Fray suppresses the *Wnk* knockdown effect and restores Sens Dll expression (Fig 1D–1D” and 1F–1F”; quantification in G, H). Conversely, co-expression of kinase dead Fray^D185A^ in *Wnk* knockdown wings significantly enhances the reduction of Sens and Dll, again consistent with a dominant negative action (Fig 1E–1E”; quantification in G,H). These epistasis analyses strongly suggest that Wnk acts though Fray during the modulation of Wnt signaling in wing development. Importantly, expression of human WNK2 rescues the effect of Wnk knockdown on Sens expression, showing that this function of WNK is conserved and that the effect is specific and not caused by an off-target effect of the *Drosophila Wnk* RNAi (Fig 1I).

**Fig 2:**
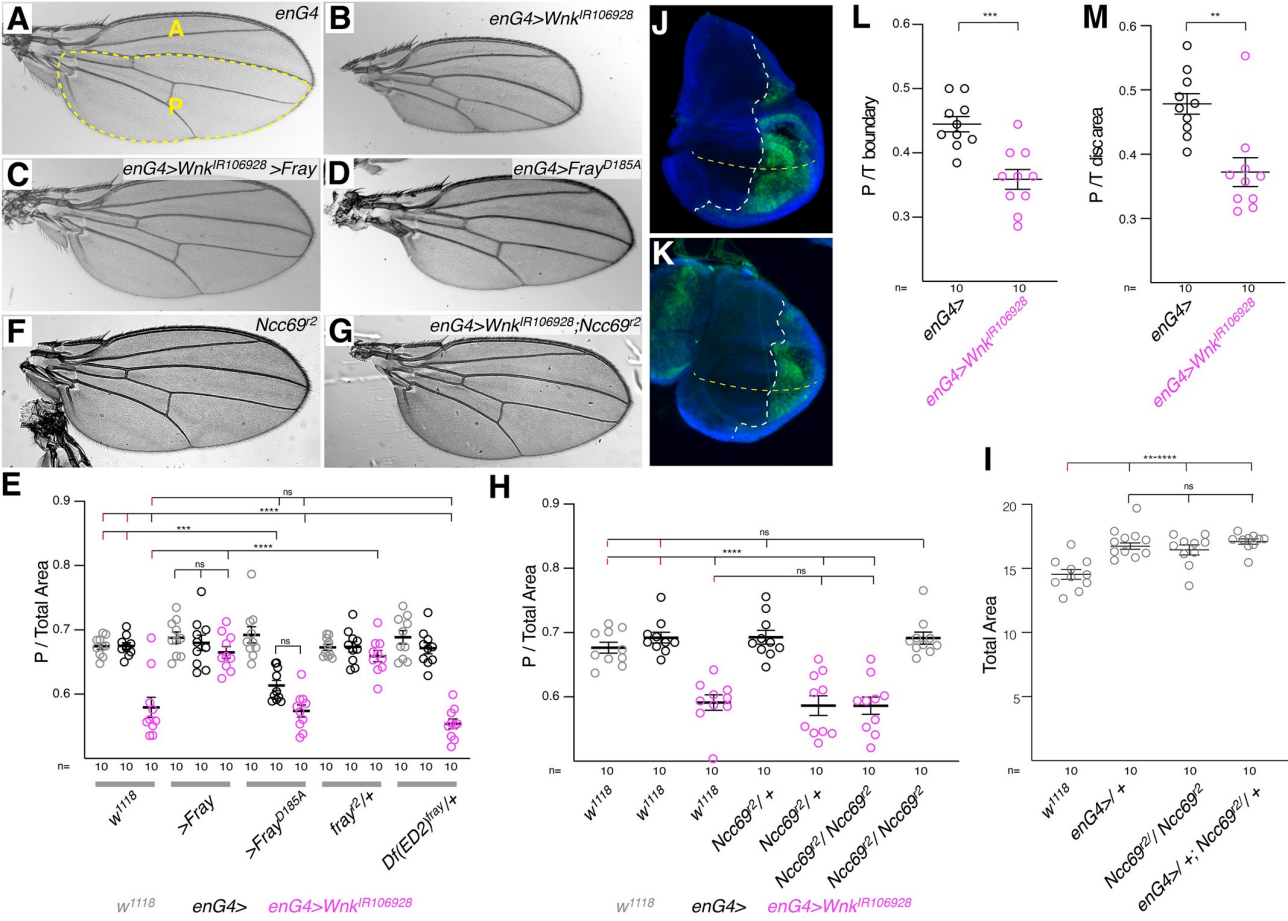
Wnk affects wing size through Fray. **(A-D)** Compared to *enGal4* control wings (A), knockdown of *Wnk* leads to a reduction of the size of the posterior compartment (approximated by the dotted yellow line) in the adult wing (B), an effect that is suppressed by co-expression of Fray (C) and phenocopied by overexpression of catalytically inactive, dominant negative *Fray*^*D185A*^ (D). A: anterior, P: posterior. **(E)** Quantification of posterior to total wing area ratio of flies indicated below graph in either *w*^*1118*^ control (grey), *en-Gal4* control (enG4>; black), or *Wnk* knockdown in the posterior compartment (*en-Gal4>Wnk*^*IR106928*^; magenta) background, respectively. Posterior wing area was calculated as outlined with dotted yellow line in (A) using L3 wing vein as approximation of the anteroposterior boundary. The *fray*^*r2*^ chromosome likely contains an unlinked suppressor, as the deletion *Df(ED2)* removing *Fray* (and additional genes) does not alter the *Wnk* RNAi phenotype. Crosses were kept at 29°C. **(F-I)** Mutation of *Ncc69* does not alter P/Total wing size in control (F) or *Wnk* knockdown (G) background; quantification in H. (**I**) Quantification of total wing size of indicated genotypes. *w*^*1118*^ flies have smaller wings than other controls or *Ncc69*^*r2*^ mutant wings. However, *Ncc69*^*r2*^ mutant wings are not smaller than *en-Gal4* controls. Crosses in F-I incubated at 29°C. **(J-M)** The effect of *Wnk* knockdown on posterior compartment size is already evident during wing development in 3^rd^ instar wing discs. Compared to control discs expressing GFP only (J), *en>Wnk*^*IR106928*^ *> GFP* discs (K) have a reduced posterior compartment measured either by the ratio of posterior to total wing width at the D/V boundary (marked by dotted yellow line; L) or by comparing posterior to total wing imaginal disc area (M). Dotted white line outlines A/P boundary. GFP-marks the *enG4* expression domain and nuclei (DAPI) are in blue. E, H, I: One-way ANOVAs (Tukey correction) P <0.0001. K, L: Student’s T-test. ****, P <0.0001; ***, P <0.001; **, P <0.01; ns, not significant.

Knockdown of Wnk or expression of dominant negative Wnk^D420A^ in the whole wing also reduces adult wing size (S1 Fig). Similarly, knockdown of *Wnk* in the posterior half of the wing only also reduces the size of the wing (Fig 2A and 2B; quantified in Fig 2E) [31]. Importantly, this is also suppressed by overexpression of Fray (Fig 2C and 2E; constitutively active Fray^T206E^ also seems to suppress, but the enlarged wings cannot be flattened for mounting and quantification and are thus not shown). Like *Wnk* knockdown, expression of dominant negative Fray^D185A^ also reduces wing size, but is not able to enhance *Wnk* knockdown in this case (Fig 2D and 2E). We did not detect an enhancement of the Wnk knockdown by heterozygous loss of Fray using a deletion of *Fray* (Fig 2E).

**Fig 3:**
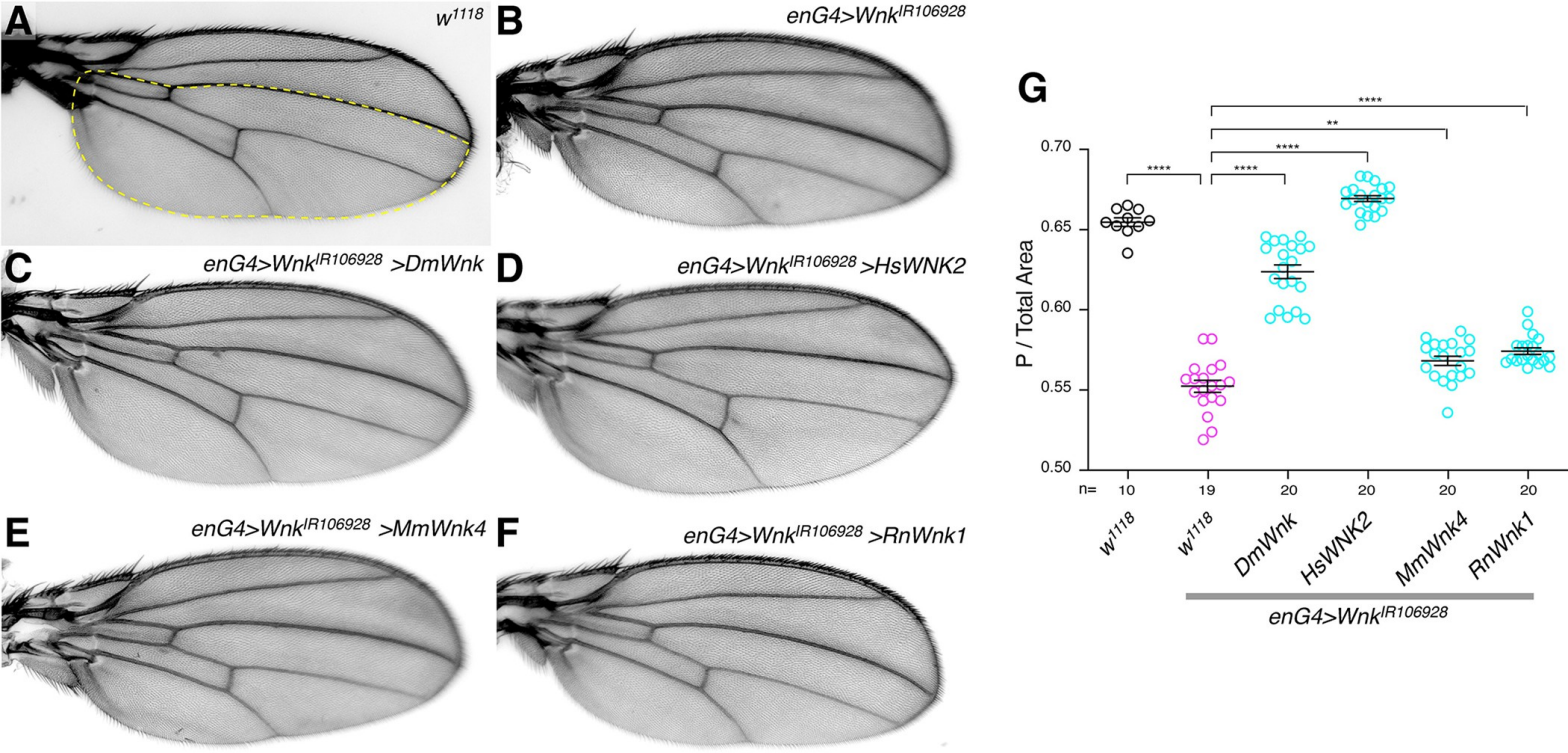
The *Wnk* knockdown wing phenotype is rescued by mammalian Wnks. **(A)** *w*^*1118*^ control wing. **(B-F)** The reduced posterior compartment size due to Wnk knockdown (B) is rescued by expression of *Drosophila Wnk* (C) and human *WNK2* (D) and partially rescued by mouse *Wnk4* (E) and rat *Wnk1* (F). Crosses were incubated at 29°C. **(G)**: Quantification of posterior compartment to total wing area. One-way ANOVA (Tukey correction) P <0.0001. ****, P <0.0001; **, P <0.01.

Removing one or both copies of the sodium-potassium-2-chloride cotransporter *Ncc69*, that mediates Wnk function for fluid secretion in the Malpighian tubules [19,20], does not modify the Wnk RNAi phenotype (Fig 2G and 2H). *Ncc69*^*r2*^ mutant wings are not smaller either (Fig 2F and 2I), suggesting that *Ncc69* either is not acting downstream of Wnk in this case, or that other ion transporters can compensate for its absence. Since WNK-SPAK/OSR1 signaling also regulates KCCs [41], we also examined interactions between loss of *Wnk* in the wing and *Drosophila kcc*. The WNK pathway generally inhibits KCCs and *kcc* knockdown thus could revert the *Wnk* knockdown effect. Although knockdown of *kcc* in the whole wing with a dsRNA construct that recapitulates *kcc* mutants [35,42] slightly decreased wing size, it did not modify the effect of Wnk^D420A^ expression, suggesting that the effects of Wnk on wing size are not mediated by KCC (S1C Fig). Compartment size reduction is not an off-target effect of the *Wnk* RNAi, as it is fully rescued by overexpression of *Drosophila* Wnk and human WNK2 and partially rescued by mouse Wnk4 and rat Wnk1 (Fig 3A–3F; quantified in Fig 3G). Thus, as in Wnt signaling in the wing (Fig 1H), mammalian WNKs can functionally substitute for fly Wnk.

**Fig 4:**
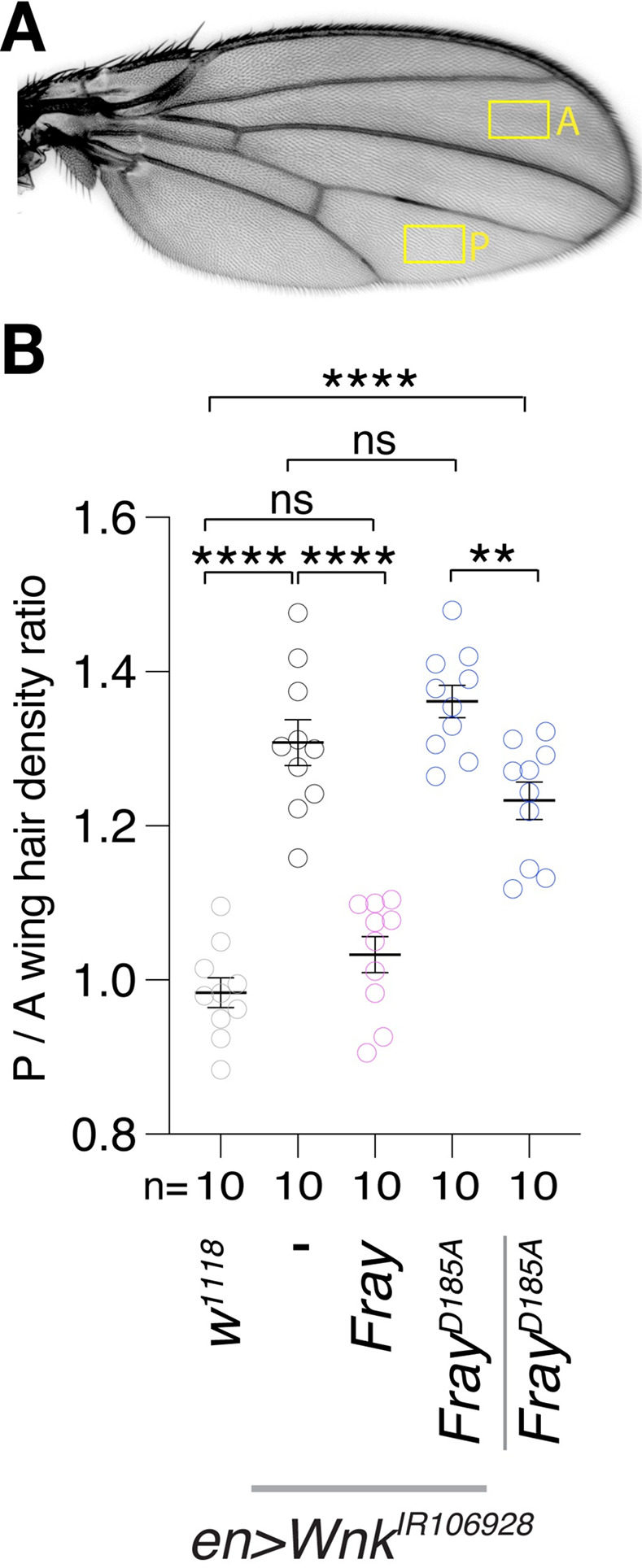
Wnk and Fray affect cell size in the wing. **(A)** Schematic of quantification of wing hair (trichome) number as surrogate for cell size in indicated fixed size areas in posterior (P) and anterior (A) compartment. **(B)** Quantification of ratio of wing hair density of the posterior versus anterior wing compartment of indicated genotypes (using ratios normalizes for variation of wing size between flies). Wnk knockdown increases posterior to anterior hair density, reflecting a smaller cell size. This phenotype is suppressed by expression of WT *Fray*, but not further enhanced by inactive *Fray*^*D185A*^. Inactive *Fray*^*D185A*^ is sufficient to increase wing hair density in the absence of *Wnk* knockdown. One-way ANOVA (Tukey correction) P <0.0001. ****, P <0.0001; **, P <0.01; ns, not significant. Only relevant comparisons are shown.

The changes in the size of the posterior compartment in *Wnk* knockdown wings are already visible during development in 3^rd^ instar wing primordial discs: Compared to control discs expressing GFP only in the posterior compartment (Fig 2J), *Wnk* knockdown discs have a smaller posterior compartment (Fig 2K; quantified in Fig 2L and 2M). The change in compartment size could either be due to changes in cell numbers or cell size. As no changes in cell death or proliferation were found in *Wnk* mutants [31], we wondered if Wnk knockdown affected cell size. To address this issue, we took advantage of the fact that each adult wing cell grows a single wing hair that points distally [43] and counted the number of hairs in a fixed area of the adult wing as a proxy for cell size (Fig 4A). Smaller cells would result in an increased number of hairs (i.e. cells) per unit area and thus alter the ratio of posterior versus anterior hair densities. Indeed, knockdown of *Wnk* (*en>Wnk*^*IR106928*^) increased wing hair density in the posterior compartment, reflected by a higher posterior to anterior hair number ratio (Fig 4B). Again, this effect is fully suppressed by expression of Fray, but not by Kinase inactive Fray^D185A^ (Fig 4B). Expression of Fray^D185A^ on its own, however, is sufficient to increase cell density (Fig 4B). These data thus suggest that loss of Wnk reduces cell size in the wing, with an overall reduction in wing size as a result. Additionally, our data show that Fray mediates the functions of Wnk kinase during the development of the wing.

### The RFSV motif of Wnk required for interaction with Fray *in vitro* is dispensable for Wnk function *in vivo*

**Fig 5:**
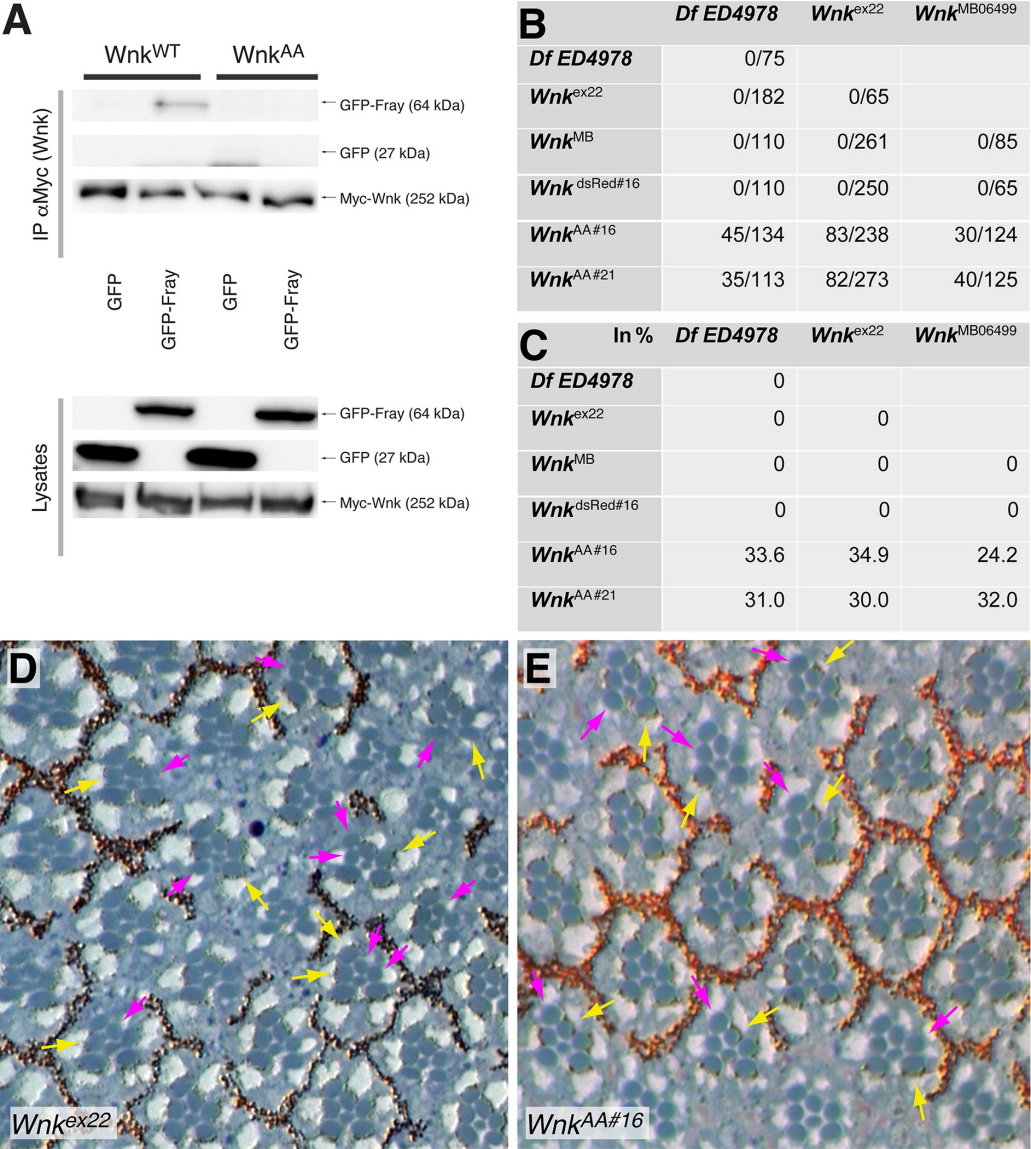
The canonical RFSV motif of Wnk is required for WNK-Fray interaction by co-immunoprecipitation but is not required *in vivo*. **(A)** In contrast to Myc-tagged Wnk^WT^, Myc-Wnk^AA^, in which the RFSV motif is mutated to AASV, is unable to immunoprecipitate GFP-Fray from lysates of transfected *Drosophila* S2R^+^ cells. Upper panels: immunoprecipitation, lower panels: cell lysates. **(B, C)** Rescue of lethality of indicated *Wnk* alleles crossed to *Df-ED4978*, which deletes the *Wnk* genomic locus (and other genes), and *Wnk*^*ex22*^ and *Wnk*^*MB*^, two *Wnk* null alleles. Two independent knock-ins, *Wnk*^*AA#16*^ and *Wnk*^*AA#21*^, which mutate the endogenous genomic *Wnk* RFSV motif to AASV, fully rescue lethality in trans to the deficiency *Df-ED4978* and the null alleles *Wnk*^*ex22*^ and *Wnk*^*MB*^. Viable mutant/ total flies are given in (B) and percentages in (C). Due to lethality of homozygous balancer chromosomes, expected Mendelian full rescue is 33%. No rescue was found with *Wnk*^*dsRed#16*^, the parental targeting integrant of *Wnk*^*AA#16*^ that still contains the dsRed marker cassette in the *Wnk* locus and represents a novel *Wnk* allele. **(D, E)** Eye sections of *Wnk*^*ex22*^ (D) and *Wnk*^*AA#16*^ (E) mosaic eyes. Wildtype rhabdomeres contain pigment granules and examples are marked by yellow arrows. In contrast, mutant rhabdomeres are identified by their lack of pigment granules and examples are marked with magenta arrows. *Wnk*^*ex22*^ mutant photoreceptors are smaller than their neighboring wildtype counterparts, a phenotype that is absent from homozygous *Wnk*^*AA#16*^ photoreceptors.

Similar to Fray mediating Wnk function in the wing during cuticle development and in Malpighian tubules in *Drosophila* [17,20,30], mammalian WNKs act through the Fray homologs OSR1/SPAK to regulate ion homeostasis [44]. For example, the cardiovascular phenotypes of *Wnk1* mutant mice are suppressed by endothelial expression of constitutively active OSR1 [45]. Mechanistically, the CCT (aka. PF2) domains of OSR1/SPAK interact with motifs that have a consensus sequence corresponding to RFx(V/I) of ion transporters and WNKs *in vitro* and in yeast two-hybrid assays [12,21,22,46]. In contrast to mammalian WNKs that often have multiple RF(x)(V/I) motifs, *Drosophila* Wnk contains a single RFSV sequence at amino acids 1794–97 (relative to Wnk-PO [47]; 1804–07 in our constructs; see schematic in Fig 7) exactly matching the canonical consensus [18,21,22]. Since *Drosophila* Wnk phosphorylates Fray [30,31] and often exerts its functions via Fray ([20,30,34,35] and above), we decided to test if the RFSV motif is required for their interaction. We thus mutated the RFSV motif to AASV (Wnk^AA^) and first tested the interaction of Wnk^AA^ with Fray by coimmunoprecipitation from lysates of cultured S2R^+^ cells *in vitro*. Compared to GFP as control, Myc-tagged Wnk immunoprecipitates GFP-Fray showing that the two proteins can interact (Fig 5A). In contrast, Myc-Wnk^AA^ is unable to bind to GFP-Fray under the same conditions (Fig 5A), showing that the RFSV motif is required for the binding of Wnk in CoIP assays.

To test the requirement *in vivo*, we made a knock-in of the same mutation in the *Wnk* locus using a Crispr approach [48] and verified it by sequencing (S2 Fig). Flies hemizygous for mutant *Wnk* null alleles or the strong hypomorph *Wnk*^*dsRed#16*^ do not survive to adulthood (Fig 5B and 5C) [30,31,49]. To our surprise, complementation analyses with two independent knock-in lines, *Wnk*^*AA#16*^ and *Wnk*^*AA#21*^, showed that both are fully viable when crossed to a deletion removing the whole *Wnk* locus, or to flies carrying null alleles of *Wnk* (Fig 5B and 5C). In addition, *Wnk*^*AA*^ mutant flies show no externally visible phenotype. This demonstrates that the RFSV motif required for binding Fray *in vitro* is dispensable for development and viability *in vivo*.

We then assessed whether additional known Wnk functions are affected in *Wnk*^*AA*^ mutants. *Wnk* mutant rhabdomeres, the light sensitive organelles in the eye, are smaller than their wildtype counterparts [31]. Unlike *Wnk*^*ex22*^ mutant rhabdomeres that are smaller (Fig 5D), mosaic analyses showed that *Wnk*^*AA#16*^ mutant rhabdomeres (Fig 5E) are of the same size as their wildtype neighbors, demonstrating that that function of Wnk does not require the RFSV motif either.

**Fig 6:**
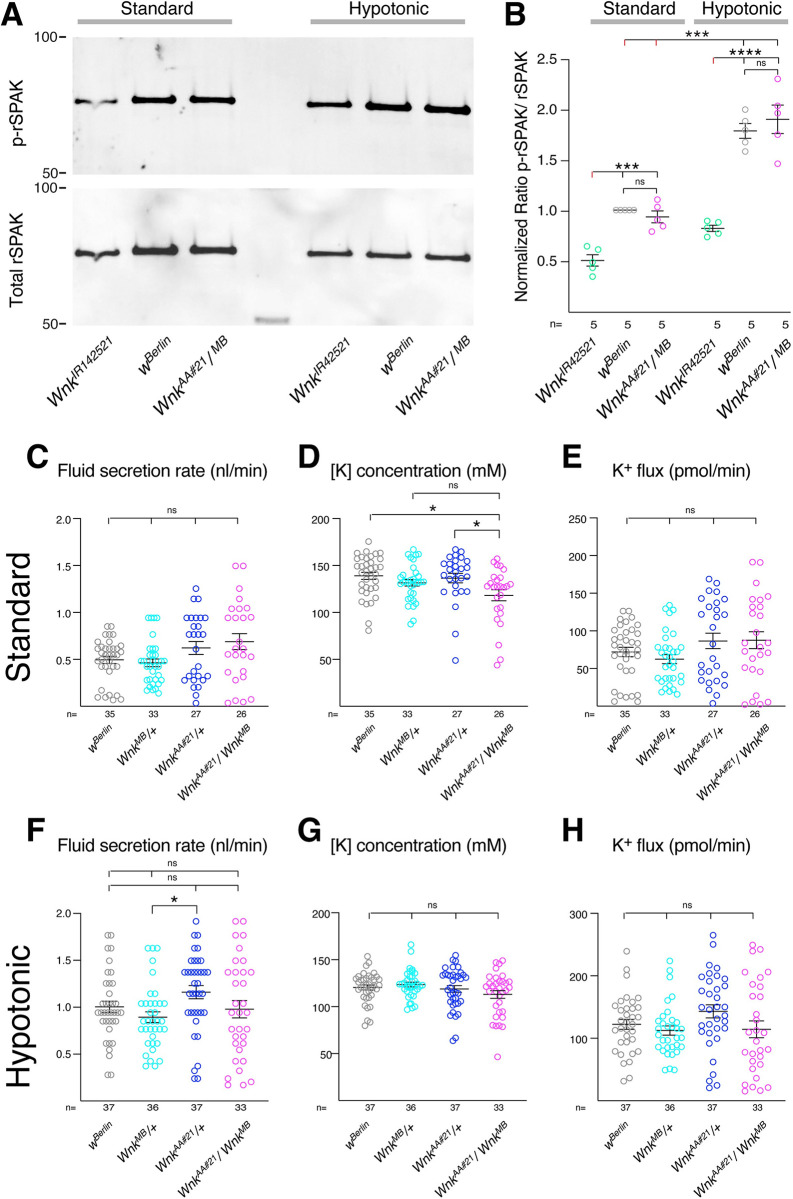
Mutation of the WNK RFSV motif does not affect WNK kinase activity or ion and fluid transport by the Malpighian tubules. **(A, B)** Wnk activity measured by phosphorylation of a kinase-dead rat SPAK^D219A^ transgene expressed in tubules in the indicated background under standard and hypotonic conditions. **(A)** Western blot of p-rSPAK (upper panel) and total rSPAK (lower panel). **(B)** Quantification of relative SPAK phosphorylation normalized to control (*w*^*Berlin*^) in standard bathing medium for each independent replicate showed the expected decrease in tubule Wnk activity upon *Wnk* knockdown and increase in hypotonic medium compared to standard bathing medium, but no difference in activity in *Wnk*^*AA*^/ *Wnk*^*MB*^ mutants compared to controls in standard bathing or hypotonic conditions. n = 5. ***, p<0.001; ****, p<0.0001, one-sample t-test to a theoretical mean of 1 (for comparisons to control in standard bathing medium) or two-sided t-test (for other comparisons). **(C-H)** Measurement of fluid secretion and transepithelial ion flux by Ramsay assays in isolated tubules in standard bathing medium (C-E) or hypotonic (F-H) conditions showed no difference in *Wnk*^*AA#21*^ */ Wnk*^*MB*^ compound heterozygotes. Potassium concentration in *Wnk*^*AA#21*^ */ Wnk*^*MB*^ was lower compared to the *w*^*Berlin*^ and *Wnk*^*AA#21*^ */ +* controls, but not compared to the *Wnk*^*MB*^ */ +* control. A significant difference was also seen between the *Wnk*^*AA#21*^ */ +* and *Wnk*^*MB*^ */ +* controls, but not between other genotypes. n is indicated for each genotype/condition (26–37 tubules / genotype). p-values for Kruskal-Wallis testing: p = 0.0908 (C, secretion, standard medium); p = 0.0094 (D, potassium concentration, standard medium); p = 0.2066 (E, potassium flux, standard medium); p = 0.0255 (F, secretion, hypotonic); p = 0.3879 (G, potassium concentration, hypotonic); p = 0.0647 (H, potassium flux, hypotonic). *, p<0.05, multiple comparisons testing.

The Malpighian tubules are the renal system of flies that regulates ion exchange and osmolarity. The tubules contain no glomeruli and are blind ended, and urine generation thus occurs though secretion of an isosmotic KCl-rich fluid from the blood across the tubules into their lumen, which is connected to the gut. Specifically, Cl^-^ ions flow though stellate cells while cations are transported through principal cells (reviewed in [17,18]). Analogous to the mammalian kidney, the Wnk/Fray cascade positively regulates Ncc69, and all of these proteins are required for normal transepithelial ion flux and fluid secretion [19,20]. For example, RNAi mediated knockdown of *Wnk* in Malpighian tubule principal cells decreases transepithelial potassium flux and fluid secretion [20]. Wnk kinase activity can be measured *in vivo* by monitoring the phosphorylation of transgenically-expressed, kinase-dead rat SPAK. The ratio of phosphorylated to total SPAK reflects WNK activity, which also was shown to be decreased upon *Wnk* knockdown in the tubule [50]. In contrast, it was shown that bathing in hypotonic medium increased Wnk activity, and Wnk, Fray-, and Ncc69-dependent ion flux in tubules [20,50]. We therefore assessed Wnk activity in standard and hypotonic bathing medium in control flies, tubule *Wnk* knockdown flies, and *Wnk*^*AA#21*^ */ Wnk*^*MB*^ flies. Transepithelial potassium flux and fluid secretion was also measured in *Wnk*^*AA#21*^ */ Wnk*^*MB*^ and in control flies heterozygous for each of the mutant alleles. As previously observed [50], Wnk activity was decreased in *Wnk* knockdown tubules and increased in controls bathed in hypotonic bathing medium. However, there were no differences in Wnk activity in *Wnk*^*AA#21*^ */ Wnk*^*MB*^ mutants compared to controls in either standard bathing medium or hypotonic medium (Fig 6A; quantification of p-SPAK to total SPAK ratio in Fig 6B). In standard bathing medium, there was no difference in fluid secretion between genotypes (Fig 6C). Potassium concentration was lower in *Wnk*^*AA#21*^ */ Wnk*^*MB*^ mutant tubules compared to two of the controls, but not compared to the *Wnk*^*MB*^ / + control, and there was no effect of any tested genotype on transepithelial potassium flux (Fig 6D and 6E). Similarly, in hypotonic medium the only statistically significant difference seen was an increase in fluid secretion rate of *Wnk*^*AA#21*^ */* + compared to *Wnk*^*MB*^ / + (Fig 6F), and there was no effect of genotype on potassium concentration or flux (Fig 6G and 6H). Thus, the Wnk RFSV motif is not required in the Malpighian tubule for kinase activity or ion transport regulation.

### The less conserved C-terminus of Wnk is essential for function *in vivo*

**Fig 7:**
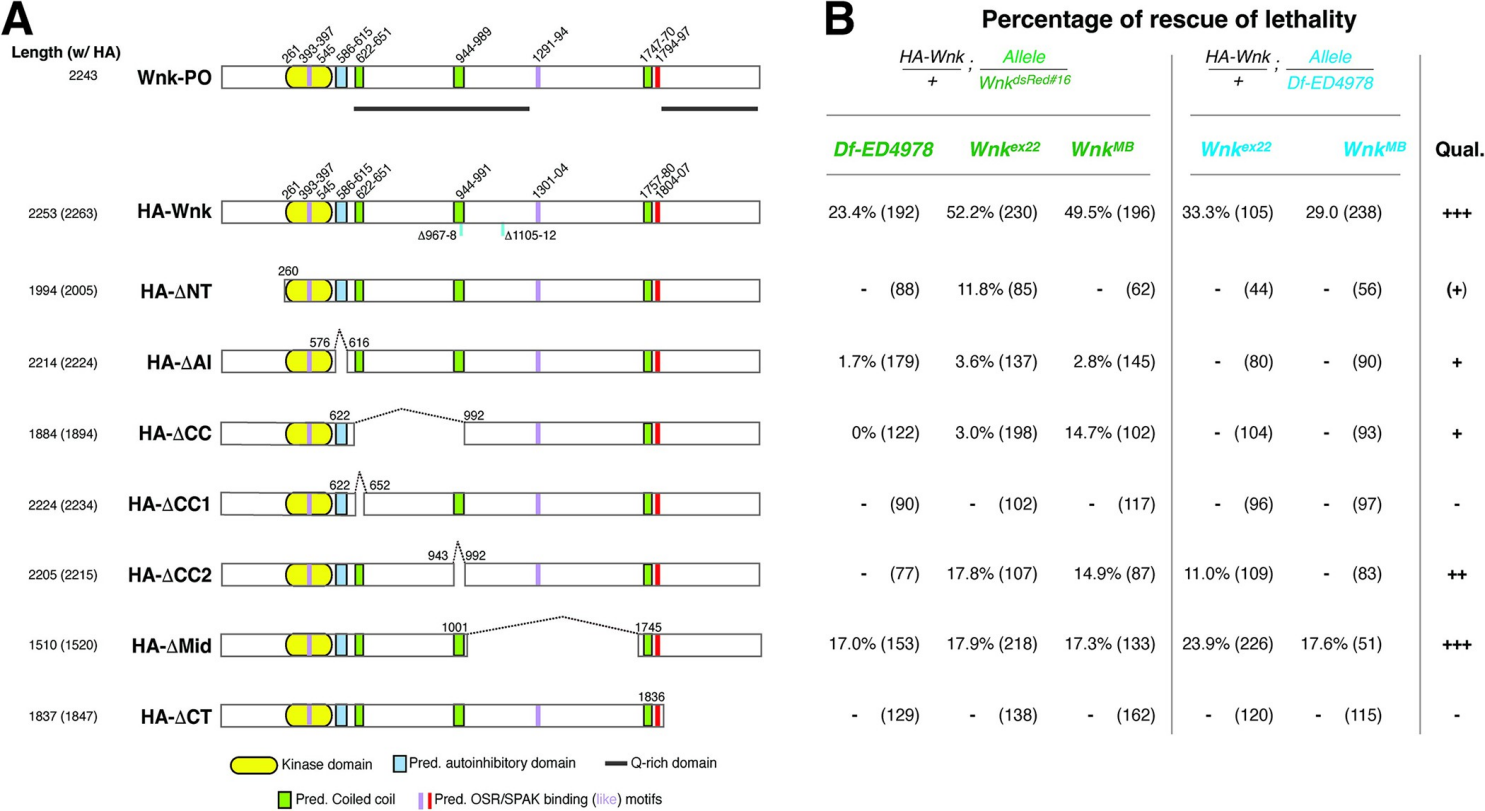
Structure function analysis of Wnk. **(A)** Schematic of full-length Wnk and deletion constructs. All constructs were N-terminally HA-tagged and expressed under the control of the α-*Tubulin* promoter. The form of Wnk amplified from cDNA is most closely related to the PO isoform in Flybase (47), the latter having two small internal deletions (light blue bars) making it 10 aa shorter. Deletion coordinates are relative to the endogenous start codon of Wnk (also see methods; total length in parentheses includes the 10 aa HA tag [w/HA]). Q-rich regions are marked by grey bars. AI: autoinhibitory domain; CC: coiled coil. **(B)** Table indicating percentage of rescue of viability of strains harboring the *Tub-Wn*k constructs of panel (A). The left part of the table shows rescue of indicated alleles in the background of the strong hypomorphic allele *Wnk*^*dsRed#16*^ (green), the right one in the background of the deletion Df-ED4978 that completely removes the *Wnk* locus (blue); see S1 Table for exact genotypes. Numbers in parentheses reflect the total flies analyzed. In all cases, Mendelian full rescue would be 16.6%, but is influenced by balancer chromosome inheritance. Qualitative (Qual.) rescue is thus indicated relative to wildtype Wnk and ‘+’ indicates rescue only of the strong hypomorph *Wnk*^*dsRed#16*^, but not of the flies carrying two null *Wnk* alleles. Note that the *Wnk*^*ex22*^ and *Wnk*^*MB*^ are null alleles, and their chromosomes contain unrelated second site lethal passenger mutations [31]. Accordingly, rescue has to be assessed in transheterozygous combinations.

WNK kinases are large proteins which are most highly conserved within the kinase domain close to the N-terminus, which is 73% identical between human WNK1 and *Drosophila* Wnk [18]. Additionally, the remainder of WNKs distal to the kinase domain contains large regions with a predicted high degree of disorder [38] interspersed with conserved motifs including an—in part predicted—autoinhibitory region adjacent to the kinase domain, coiled coil areas, and RF(x)V/I motifs (Fig 7A for schematic of *Drosophila* Wnk) [18]. Additionally, *Drosophila* Wnk also contains regions that are highly enriched in glutamines (Q-rich; Fig 7A) [38,51]. The functional relevance of most of these domains has never been studied. We thus took an extensive genetic rescue approach using Wnk truncations and internal deletions that were expressed under the control of the ubiquitously expressed *αTubulin* promoter in various *Wnk* mutant backgrounds (Fig 7 and S3 Fig for expression levels). In particular, we deleted the N-terminus upstream of the kinase domain (HA-ΔNT), the predicted autoinhibitory domain just C-terminal to the kinase domain (HA-ΔAI), and the C-terminus after the RF(x)V/I motif containing the second Q-rich, disordered area (HA-ΔCT). Additional deletions span regions of the predicted coiled-coil domains 1&2 alone and together with the region in between (HA-ΔCC1, HA-ΔCC2, and HA-ΔCC, respectively) and a large area in between CC2 and CC3 (HA-ΔMid). Rescue transgenes then were crossed into five different *Wnk* mutant backgrounds to assess the ability of the transgene to rescue the lethality due to loss of *Wnk*. These mutant backgrounds contained combinations of the strong hypomorphic allele *Wnk*^*dsRed#16*^, the null alleles *Wnk*^*ex22*^ *and Wnk*^*MB*^ [31], as well as the *ED4978* deletion that lacks the entire *Wnk* locus (and additional flanking genes; complete genotypes in S1 Table). In all cases the mutant alleles were in trans to one another to avoid effects from unrelated second site passenger mutations. Full rescue of lethality was found for wildtype HA-Wnk (Fig 7B) in all combinations assessed. Similarly, rescue was obtained for HA-ΔCC2 and HA-ΔMid, suggesting that the 2^nd^ coiled coil domain and a large part of the likely disordered middle part of the C-terminus, including a KFDI sequence related to the RF(x)V/I motif [52], are dispensable for Wnk function. Forms of Wnk lacking the N-terminus, the autoinhibitory sequence and the region from CC1 to CC2 still have partial activity, as they rescue lethality of Wnk^dsRed#16^ to various extents, but not a null background (*Wnk*^*ex22*^*/Df-ED4978* or *Wnk*^*MB*^*/ Df-ED4978*). No rescue was found for Wnk forms lacking CC1, or, critically, the C-terminus distal to the RFSV motif. Taken together, these results show that, while a large central portion of Wnk is not essential for viability, other regions of Wnk, in spite of being poorly conserved at the primary sequence level, nevertheless are essential for its function (see also discussion).

## Discussion

WNK kinases generally act through their downstream kinases OSR1/SPAK/Fray to regulate the activity of NKCCs and KCCs in an opposing manner, thereby controlling ion homeostasis and cell volume [4–7,17]. However, it was unknown to what extent Fray was involved in the modulation of Wnt signaling in *Drosophila*. Here, we showed that the reduction of the Wnt signaling targets Sens and Dll by knockdown of *Wnk* in the wing during development are suppressed by the overexpression of constitutively active Fray, clearly indicating that Fray acts downstream (or in parallel) of Wnk (Fig 1). This is consistent with a requirement of OSR1 and SPAK for Wnt signaling in mammalian cell culture [31]. Initially, Wnk has been shown to act upstream or at the level of the Wnt signaling adapter protein Dishevelled (Dsh), as knockdown of *Wnk* reduced Dsh phosphorylation [31]. More recently, it has been shown that WNK kinases affect Wnt signaling in more complex ways, as they also attenuate an interaction between the transcription co-factor β-Catenin and the E3 ligase GID (glucose-induced degradation deficient) complex involved in β-Catenin degradation in cultured human cells, resulting in a net positive effect on Wnt signaling (33). Most likely, the stabilizing effect of WNKs on β-Catenin is mediated via OSR1/SPAK as well, as one of the two tested small molecule inhibitors that prevent a WNK/SPAK interaction also led to reduced Wnt signaling output [33]. We furthermore showed that the reduced posterior compartment size of adult wings upon *Wnk* knockdown is a specific phenotype due to loss of Wnk, as it can be rescued by several mammalian WNKs (Fig 3). Additionally, this phenotype, which already is visible during larval development, is also suppressed by expression of Fray and phenocopied by dominant-negative Fray (Figs 2 and S1). Using wing hair density as a surrogate for measuring cell size, we showed that this effect is due to a reduction of cell size (Fig 4). Our data thus show that the known functions of Wnk in wing development are exerted via Fray. In contrast, we found no effect of the sodium-potassium-2-chloride cotransporter Ncc69 that acts downstream of Wnk and Fray in the Malpighian tubules [19] on wing development, nor was knockdown of *Kcc* able to suppress the effect of loss of Wnk. This indicates that they either are not required for those Wnk functions, or that they act redundantly with other transporters. Nkcc83 would be a candidate for such a redundancy.

OSR1 and SPAK were originally identified in two-hybrid screens with fragments of several NKCCs and KCCs. The interaction was mapped to the conserved OSR1/SPAK C-terminal domains (CCT) that were shown to bind core RF(x)V/I consensus motifs on ion transporters and WNKs ([22,52], reviewed in [21]). Double mutation of RF to AA abolished the interactions in yeast [52] and, similarly, mutating both putative RF(x)V/I motifs in *C*. *elegans* WNK-1 prevents (most) binding to GCK-3, the worm OSR1/SPAK/Fay homolog [53]. Since we showed that Wnk functions in flies depended on Fray, we tested the requirement of the RFSV motif of *Drosophila* Wnk. Mutating RF to AA in the single canonical RF(x)V/I motif in *Drosophila* Wnk abolishes the interaction between Wnk^AA^ and Fray in CoIP assays (Fig 5A). However, and in contrast to *C*. *elegans*, where mutating both motifs prevents rescue activity [53], to our surprise, the knock-in of the Wnk^AA^ mutation *in vivo* is viable with adult flies showing no overt phenotypes. Significantly, Malpighian tubule potassium concentration, ion flux and fluid secretion, functions known to depend on Fray [20], are also normal. The most plausible explanation for this would be additional proteins present *in vivo* may stabilize an interaction between Wnk and Fray. One such candidate could be Mo25, the fly homolog of mouse MO25α and β (mouse protein 25 α/β) which are redundant proteins that bind to OSR1/SPAK and strongly stimulate their activity [41,54]. In *Drosophila*, *fray* and *mo25* mutants show similar phenotypes in the asymmetric division of embryonic neuroblasts, and, importantly, their co-overexpression is required to generate gain of function phenotypes in that context [55]. However, Fray and Mo25 do not co-precipitate from embryonic extracts [55]. An alternative explanation for normal function of Wnk^AA^ could be that Wnk contains a different RF(x)V/I-like motif that would have to be functional *in vivo* but not *in vitro*. Wnk has one KFDI sequence where the lysine would replace arginine as the positively charged amino acid (amino acids 1311–14; Fig 7). Not only is this motif not sufficient for interaction with Fray in the CoIPs (and thus not redundant with the RFSV motif), but those amino acids are also within the large middle domain that is dispensable for Wnk function on its own (Fig 7) and none of the known functional RF(x)V/I motifs contain a Lysine at position 1 [21,22]. Additionally, in silico studies have shown that a Lys at this position would be less favorable [56]. More recently, a non-canonical R(x)F(x)V/I sequence has been identified as a natural variant of the RF(x)V/I motif in several inwardly rectifying K^+^ channels [57], a version of which can be found in Wnk [R(Q)F(P)I] at position 393. This motif is in the middle of the kinase domain, but despite high overall sequence identity between *Drosophila* and mammalian WNK kinase domains (e.g. 73% to huWNK1 [18]), mammalian WNKs do not contain a R(x)F(x)V/I motif at this location. Furthermore, mutation of an RF(x)V motif at a distinct position within the WNK1 kinase domain did not impair WNK1 kinase activity [58]. Finally, no other known motif contains a Pro upstream of the I/V [21]. Therefore, although we cannot definitively rule out roles for these non-canonical motifs, existing evidence suggests that they are less likely to be redundant in mediating the Wnk-Fray interaction. To our knowledge, this therefore is the first time that an OSR1/SPAK/Fray-dependent function of a WNK kinase does not require a RF(x)V/I motif *in vivo*, and we thus suggest that the *in vivo* Wnk-Fray interaction is stabilized by (an) unknown factor(s) that will have to be identified in future experiments.

Many domains of the large WNK kinases have not been studied as completely as the kinase domain. We therefore performed a stringent, rescue of lethality-based structure function analysis to assess the requirement of several such domains. Mutation of the so-called autoinhibitory domain (AI, aka. CCTL1 or CCT-like domain 1) adjacent to the kinase domain and conserved between mammalian and *Drosophila* WNKs increases kinase activity [59,60], but the physiological relevance of this was uncertain [21]. Here, our analysis showed for the first time that loss of the AI domain significantly reduces Wnk function *in vivo*. We also found that a large region of poorly conserved amino acids between the second and third predicted coiled-coil (CC) domains is dispensable for Wnk function, as is the CC2 itself (ΔMid; Fig 7). Interestingly, this region is less Q-rich than the other parts of the C-terminus. Wnk retains partial activity when the Q-rich region from CC1 to CC2 is deleted, although deletion of CC1 alone abolished rescue. It is possible that the ΔCC1 deletion induces unfavorable structural changes indirectly influencing Wnk activity that are not occurring in the context of the larger CC1 to CC2 deletion.

WNKs are activated by hypotonicity as well as hypertonicity, the latter also leading to a redistribution of WNKs to punctate structures in cells [18,61]. Hyperosmotic shock results in an acute cellular shrinking that is quickly compensated for by ‘regulatory volume increase’, with a rapid cytosolic increase of Na^+^, K^+^, and Cl^-^ ions mediated by, amongst others, WNK kinase pathway stimulation of NKCC [61–63]. How WNK is activated under those conditions has been enigmatic, as high intracellular Cl^-^ concentrations directly inhibit WNK kinase [64]. Interestingly, it has recently been shown that the WNK puncta induced in cell culture within seconds by hypertonic stress are membraneless organelles (MLOs) that form via phase transition [38]. These MLOs also recruit OSR1 and SPAK [38], and it has been postulated that molecular crowding in condensates allows WNK activation in spite of unfavorable ion concentrations in the cytoplasm [38]. A key region required for MLO formation in culture was mapped to a disordered domain at the C-terminus of human WNK1 and *Drosophila* Wnk [38]. Our data now show that this domain is indeed essential for Wnk function *in vivo* (HA-ΔCT in Fig 7). The region following the kinase domain, corresponding approximately to the CC1 to CC2 region of *Drosophila* Wnk, also mediates some of the phase separation behavior of both WNK1 and *Drosophila* Wnk, but to a lesser extent than the C-terminal region, and its deletion impaired the ability to phosphorylate OSR1/SPAK in response to hypertonic stress [38]. Consistent with these findings, deletion of this domain of *Drosophila* Wnk partially impairs its ability to rescue Wnk function *in vivo*. Interestingly, SPAK and OSR1 are recruited into WNK1 condensates in response to hypertonic stress [38]. The molecular determinants for this are unknown, but this could represent an additional mechanism for WNK-SPAK/OSR1/Fray interactions (e.g. in the absence of the RSFV motif). Our functional domain analyses *in vivo* thus provide strong *in vivo* support of recent data from cell culture.

In summary, we demonstrate that Wnk signaling via Fray regulates the Wnt targets Dll and Sens in wing development, as well as wing cell size. Despite this, the only canonical RF(x)V/I motif in the Wnk C-terminus is required for WNK-Fray interactions in CoIP assays, but is dispensable *in vivo* for *Drosophila* development, viability, and ion flux and fluid secretion in the Malpighian tubules. In contrast, our structure function analysis emphasizes the importance of the less well conserved Wnk N- and C-termini. Our work thus extends understanding of different domains of WNKs in developmental and physiological processes.

## Material and methods

### Fly husbandry and strains

Flies were maintained at 25°C on a 12:12 hr. light/dark cycle on standard cornmeal/yeast/molasses diet unless mentioned otherwise. *P[GawB]Bx*^*MS1096*^ (*MS1096* on X chromosome; RRID:BDSC_8860), *Df (3L) ED4978* (*Df-ED4978*; RRID:BDSC_8101) and *Herm(3XP3-ECFP*, *a-Tub piggybac10)M6* (RRID:BDSC_32070) were from the Bloomington *Drosophila* stock center. *kcc*^*IR101742*^ was from VDRC and recapitulates *kcc* loss-of-function mutant alleles [35,42]. *c42-GAL4* was a kind gift of Dr. J. Dow (Glasgow, UK) [65]. The following lines were as described: *enGal4>UAS-Wnk*^*IR106928*^, *Wnk*^*IR42521*^ (RRID:BDSC_42521),*Wnk*^*ex22*^ *FRT80* (*Wnk*^*ex22*^; RRID:BDSC_99482; see also S2 Table), *Wnk*^*MB06499*^ *FRT80* (from here on: *Wnk*^*MB*^) [31]; *w*^*Berlin*^, *UAS-Wnk*^*D420A*^ and *w*^*Berlin*^, *UAS-Fray*^*T206E*^ (RRID:BDSC_99479) [20]; *Ncc69*^*r2*^ [66]*; fray*^*r2*^ [67]; *UAS-rnSPAK*^*D219A*^ [50]. The efficiency of *UAS-Wnk*^*IR106928*^ (VDRC) and *UAS-Wnk*^*IR42521*^ have previously been assessed in Malpighian tubules and shown to reduce *Wnk* transcript levels at least to 40% when expressed under control of *c42-Gal4* in a subset of tubule cells [20,50]. *Ncc69*^*r2*^ and *fray*^*r2*^ were a kind gift from Dr. W. Leiserson (Yale) [66,67]. Mammalian WNK constructs and transgenic flies were described in [68]. *UAS-Wnk*^*D420A*^, *MS1096*, *kcc*^*IR101742*^, *c42-GAL4*, *UAS-Wnk*^*D420A*^, *UAS-Fray*, *UAS-Fray*^*T206E*^, *UAS-Fray*^*D185A*^, *UAS-rnSPAK*^*D219A*^ and *UAS-mammalian WNK* transgenes were outcrossed for 5 generations to the Rodan laboratory *w*^*Berlin*^, as was *Wnk*^*MB06499*^ (without FRT) used in Malpighian tubule assays.

*Antibodies, immunohistochemistry, wing size measurements, and eye sectioning*

For immunostaining, third instar larvae were dissected in ice-cold PBT (0.1% Triton) and fixed in 4% paraformaldehyde in PBS for 20 minutes at room temperature and stained as described [69] keeping the blocking step at 15 minutes and performing the whole procedure on the same day. Antibodies were as follows: mouse anti-Ptc (Apa1; 1:100; DSHB) and guinea pig anti-Sens (1:1000; a kind gift of H. Bellen). Rabbit anti-Dll antibody was generated by ThermoFisher Custom Antibodies against the peptide EFPPTGLSPPTQAPWDQKPH (residues 250–269 of Uniprot entry P20009, but present in all annotated isoforms) at New Mexico State University and was used at 1:100. FITC anti-rabbit, 568 anti-guinea pig, and 647 anti-mouse secondaries were from Life Sciences and used at 1:300. Discs were mounted in 20 μl Vectashield (Vector Labs H-1000). Confocal images were taken on a Nikon spinning disc confocal microscope at 20x magnification at the Albert Einstein Imaging facility. 11 sections of an image stack of discs imaged under identical settings were combined into a single image using the ‘sum’ projection of Fiji/ImageJ [70]. Average staining intensities of anterior and posterior ROIs of equal size (sized to fit ventral Sens signal on the anterior as outlined with dotted yellow boxes in Fig 1A) were quantified using Fiji/ImageJ [70] and analyzed using GraphPad Prism (Versions 10; GraphPad Software, La Jolla, CA). Throughout, graphs represent means ± SEM unless noted otherwise. *enGal4>UAS-GFP* discs were fixed, washed in PBT and directly imaged after mounting in fluoromount-G on a Zeiss Axio-imager at 20x magnification.

For adult wing analysis, wings of females only were assessed to avoid complications with dimorphisms [71] and with the X-chromosomal driver *MS1096*. Cut-off wings were incubated in 0.1% Triton-X100 in PBS for at least one hour and subsequently mounted in 80% glycerol in 1xPBS as described [72]. For the quantification of wing compartment ratios in enGal4 experiments, full and posterior compartment wing areas of images takes at 5x magnification were traced using the free-hand and polygonal tracing tools of Fiji/ImageJ. Note that the A/P boundary running in between wing veins L3 and L4 was approximated for reproducibility by using L3 as a clearly identifiable landmark. To quantify the wing size in the *MS1096-Gal4* experiments, wings were approximated by landmarks outlined in S1 Fig (see legend there). Wing area was then quantified using the Olympus CellSens software. To quantify wing hair densities, wing images were taken at 10x magnification with a 0.63x camera adapter and an open field aperture to reduce focal depth. Hairs were then counted in 200 μm x 120 μm areas (corresponding to 199 x 119 pixels) in the posterior ‘D’-wing cell and anterior ‘B’-wing cell [73] (indicated with yellow rectangles in Fig 4A). The ratio of posterior to anterior hair numbers per area (hair density) was then calculated to account for variations in overall wing size. For genetic interactions in wings at 29°C, fly crosses were incubated at 25°C for 2 days prior to shifting crosses to 29°C. Adult eye clones induced by ey-FLP were embedded and sectioned as described [74].

### Molecular biology and transgenics

All PCR fragments were amplified with Cloneamp HiFi PCR (Takara) and sequence verified. Final plasmids are available from Addgene (S2 Table). *UAS-Fray* (RRID:BDSC_99477) and *UAS-Fray*^*D185A*^ (RRID:BDSC_99478) transgenics were generated analogously to [20] by cloning wild-type and mutated Fray cDNAs generated in that study into pUASg.attB (a kind gift of Dr. K. Basler, University of Zürich) [75]. DNA was injected into *y*^*1*^*M[vas-int*.Dm]ZH-2A w^*^; M[3xP3-RFP.*attP’]ZH-22A* and transformants selected based on eye color. The presence of the correct insert confirmed by genomic DNA PCR and sequencing.

pAttB-Tub was made by cloning the *Tubulin* promoter as EcoRI/ NheI fragment of pCaspTubPA [76] into the EcoRI/ XbaI sites of pAttB (a kind gift of Dr. K. Basler, University of Zürich) [77].

Note that we use the start codon of Wnk as in [30,31] corresponding to transcripts related to isoforms RO ([47] and personal communication with Flybase curators (FBrf0226488)). To clone HA-Wnk, RNA was prepared using Trizol (Invitrogen) from *w*^*1118*^ flies and oligo-dT primed cDNA was synthesized using the SuperScript IV First-Strand Synthesis System (Invitrogen) according to the instructions of the manufacturers. Wnk was amplified with primers Wnk-RO-Fw-NotI-HA and Wnk-RO-Rv-Avr (S3 Table for all oligonucleotides) and cloned into pCR8-GW-Topo. After sequence verification, HA-Wnk was cloned as NotI/ AvrII fragment into the NotI/ XbaI sites of pAttB-Tub (pAttB-Tub-HAWnk; fly strain RRID:BDSC_99483). pAttB-tub-WnkΔNT was made by amplifying pAttB-Tub-HA-Wnk with primers WnkRO-Nterm-Del-Fw and WnkRO-Nterm-Del-Rv and closing the fragment using Gibson ligation (NEB; PCR products used for Gibson cloning were DpnI digested prior to gel purification). Cloned Wnk fragments contain two small insertions of 2 (QQ) and 8 amino acids (QQQSMVQQ) compared to the Wnk-RO sequence in Flybase at positions indicated in Fig 7. Deletions ΔAI, ΔCC, ΔCC1, and ΔCC2 were made by Gibson ligation of pAttB-Tub-Wnk digested with StuI and XbaI as backbone and two PCR products amplified with WnkRO-StuI-Fw as common forward and WnkRO-XbaI-Rv as common reverse primer, respectively, in combination with the following deletion specific reverse and forward primers: ΔAI: WnkRO-Del-AI-Rv and WnkRO-Del-AI-Fw; ΔCC: WnkRO-CCdelete-Rv and WnkRO-CCdelete-Fw; ΔCC1: WnkCC1del-Rv and WnkCC1del-Fw; ΔCC2: WnkCC2NewDel-Rv and WnkCC2NewDel-Fw. pAttB-Tub-WnkΔMid was made analogously, digesting pAttB-Tub-Wnk with StuI and NdeI as backbone and two PCR products amplified with WnkRO-StuI-Fw and WnkRO-mid-del-Rv, and WnkRO-mid-del-Fw and WnkRO_NdeI_Rv, respectively. pAttB-Tub-HAWnkΔCT was made by inserting annealed, unphosphorylated WnkRO-StopAvrII-Fw and WnkRO-StopAvrII-Rv oligos into the SfiI site of pAttB-Tub-HAWnk. DNAs were integrated into attP40 on 2L to reduce effects of chromatin accessibility (injections were done by Rainbow Transgenics, CA).

pENTR3c-Wnk-RO was assembled from N-terminal (to SalI) and C-terminal (from SalI) PCR fragments amplified with primers pENTR3CWnkRO_Fw, pENTR3CWnkORF_N_midR, and pENTR3CWnkORF_C_midFW, pENTR3CWnkRO_Rv, respectively, using oligo-dT primed cDNA as above using Gibson ligation into pENTR-3C-Dual (Invitrogen). Full-length Wnk was then made by combining the appropriate SalI/ PvuI fragments. pENTR3c-Fray was made by cloning Fray as BamHI/ XhoI fragment of pGex4T1-Fray [31] into the corresponding sites of pENTR3c-Dual. Actin promoter driven Wnk and Fray for expression in S2R^+^ cells were made by Gateway cloning (Invitrogen) of pENTR3c-Wnk-RO and pENTR3c-Fray into pAMW and pAGW destination vectors of the *Drosophila* Gateway vector collection, respectively. The RFSV motif in Wnk was mutated to AASV in pAMV-Wnk by cloning annealed oligos wnk_RFtoAA_FW and wnk_RFtoAA_RW into its BssHII /SfiI sites to give pAMW-WnkAAxV (also introducing a silent NheI site).

To mutate the RFSV motif of Wnk to AASV *in vivo*, we followed the scarless strategy adapted from [48]. Briefly, left and right homology arms were amplified from genomic DNA of *nos-Cas9; attP40* (Rainbow Transgenics) flies using RFxV-leftArm_for_hom, and RFxV-leftArm_mut_rev, and RFxV-RightArm_for_hom and RFxV-rightArm_rev_hom (S3 Table), respectively. Gibson ligation was used to combine the arms with both pSHD-dsRed fragments released upon digestion with SapI and AarI. Note that this introduces the AA mutation of RF, a silent NheI site for easy identification of the allele (S2 Fig), and mutates the PAM site preventing re-cleavage of the targeted allele (changes contained in RFxV-leftArm_mut_rev). The final plasmid contains the homology arms flanking a 3P3-dsRed cassette expressing RFP in the eye that mimics a pBac transposon. A suitable gRNA was identified using ChopChop [78]. pCFD-Wnk188 expressing the gRNA under the U6 promoter was made by cloning annealed oligos pCFD3_wnk_gRNA_sense and pCFD3_wnk_gRNA_anti into the BbsI site of pCFD3 [79]. Both plasmids were co-injected into *nos-Cas9 attP40* embryos (Rainbow Transgenics, CA) and two independent integrants, *Wnk*^*dsRed#16*^ (RRID:BDSC_99480) and *Wnk*^*dsRed#21*^ were further characterized by sequencing the introduced mutation, homology arms, and breakpoints upon PCR amplification. *Wnk*^*dsRed*^ integrants reflect novel *Wnk* alleles and truncate Wnk 20 AA after the AA mutation (that are followed by 8 ectopic amino acids prior to a stop codon). They likely are strong hypomorphs (see complementation tests in Figs 5 and 7). The dsRed cassette was then excised *in vivo* using pBac transposase *Herm (3XP3-ECFP*, *a-Tub piggybac10)M6* and selecting against red and cyan fluorescence to give alleles *Wnk*^*AA#16*^ (RRID:BDSC_99481) and *Wnk*^*AA#21*^. Proper excision was confirmed by sequencing upon amplification of the mutated area including the homology arm breakpoints with primers Wnk-cr_seq-Left-arm-Fw and Wnk-cr_seq-Right-arm-Rv. Sequence traces of mutated region is shown in S2 Fig. *Wnk*^*AA#16*^ FRT80 was made by recombining *Wnk*^*dsRed#16*^ onto FRT80 prior to removing the pBac-dsRed cassette.

### Immunoprecipitations and biochemistry

For co-immunoprecipitations, 6.3*10^6^ S2R^+^ cells (*Drosophila* Genome Resource Center Isolate #150) were seeded on 60mm plates and transfected with 2.1 μg of each of the indicated plasmids using Effectene (Qiagen) according to the instructions of the manufacturer. Immunoprecipitations were done as described using 1 μg anti-Myc (Santa Cruz 9E10; #SC-40) [80]. After separation by SDS-PAGE, proteins were transferred onto PVDF membranes and probed using standard procedures with mouse anti-GFP (1:1000; Roche # 11814460001) and α-Myc (1:1000). HRP conjugated secondary goat anti-mouse antibody (Jackson Immunoresearch Lab #115-035-003) was used at 1:10,000.

For rescue construct expression level tests, 15 fly heads were suspended in 100 μl 1x Laemmli buffer (2% SDS, 10% glycerol, 5% 2-mercaptoethanol, 0.002% bromophenol blue, 62.5 mM Tris HCl, pH 6.8) and boiled for 5 min at 95°C [81]. After homogenization with a motor pestle (DWK Life Sciences-Kimble, 749540–0000), lysates were boiled again and centrifuged twice for 10 min at 20,000 g at room temperature. After each centrifugation, 90% of the liquid phase was removed avoiding lipids floating on top. Upon separation of the proteins on an 8% SDS-PAGE, proteins were transferred to PVDF and Westerns probed with rat anti-HA (Roche # 11867423001) at 1:1000 and anti-TUBA/αTubulin (Sigma, T5168; 1:10,000) as loading control.

HRP signal was detected using ECL (Pierce # PI32106) and a G:Box Chemi-XX6 gel documentation system (Syngene). Tif flies were quantified with Multi Gauge 3.0.

### Malpighian tubule secretion assays

Female flies were collected within 1–2 days of eclosion and kept for 3 days before tubule dissection. For Western blots, 15 pairs of anterior Malpighian tubules expressing kinase-dead rat SPAK^D219A^ under the control of *c42-GAL4* in the indicated mutant background were dissected from adult females in *Drosophila* saline [50]. Tubules were transferred to 300 μl of standard bathing medium (SBM: 1:1 ratio of Schneider’s *Drosophila* Medium (Thermo Fisher #21720001) to *Drosophila* saline) for 1 h in a 9-well Pyrex dish covered by Parafilm to prevent evaporation. After 1 h equilibration, SBM samples were immediately lysed in 30 μl of 2X Laemmli sample buffer (BioRad). For hypotonic medium samples, an additional 80 μl of distilled water (to generate hypotonic medium) were added to each well. Tubules were allowed to bathe for an additional 30 min at room temperature. Tubules were then transferred to 30 μl 2X Laemmli sample buffer (BioRad). 20 μl of lysate was used to detect phosphorylated SPAK (rabbit anti pSPAKSer373/pOSR1Ser325 [Millipore #07–2273]; 1:1,000) and total SPAK (mouse anti-STK39 2E10; GeneTex #GTX83543) simultaneously by western blotting [50] using the following secondary antibodies at 1:10,000 dilution: Azure Spectra Florescent goat-anti-mouse IR700 (Azure Biosystems #AC2129) and Azure Spectra Florescent goat-anti-rabbit IR800 (Azure Biosystems #AC21034). Protein bands were visualized using a c600 Azure Biosystems instrument and quantified in ImageJ by manually outlining the bands and subtracting background pixel intensities from a nearby region. To account for day-to-day variability in the western blotting procedure, the p-SPAK/ total SPAK ratio of mutants in each Western blot was normalized to the corresponding control. The antibodies to p-SPAKSer373/pOSR1Ser325 and STK39 were previously validated in the Malpighian tubule [50] and were re-validated prior to use in these experiments using Malpighian tubules that were not expressing SPAK.

For Ramsay secretion assays, Malpighian tubules were dissected from adult females in *Drosophila* saline and transferred to wells containing SBM or hypotonic medium, and potassium flux and fluid secretion after two hours was measured as previously described [50,82].

## Supporting information

S1 Fig Knockdown of *kcc* does not alter the wing size reduction caused be the expression of kinase inactive, dominant-negative *Wnk*^*D420A*^ expressed in the wing pouch. **(A)** Schematic outlining the wing area quantified using landmarks. #1, 3: beginning and end of wing vein L2; #4, 5 mark the ends of L3 and L4, respectively; #2, 7: wing margin crossing points of hypothetical extension of posterior cross vein (PCV); #6: margin crossing of a line extended from #1 to the beginning of the PCV on L4; #8: margin crossing of a line extended from #4 through the intersection of the PCV with L5. **(B, C)** Quantification of wing sizes of indicated genotypes. (B) Knockdown of *Wnk* in the whole wing pouch by *MS1096-Gal4* causes a reduction of the total wing size (B), as does expression of dominant-negative Wnk^D420A^ (C). Compared to controls, knockdown of *kcc* in the wing pouch slightly reduces wing size on its own (C), but does not affect the size reduction due to *Wnk*^*D420A*^. One-way ANOVA (Tukey correction) P <0.0001. ****, P <0.0001; **, P <0.01; ns, not significant. Only relevant comparisons are shown.
(TIF)

S2 Fig Sequence verification of the knock-in *Wnk*^*AA*^ alleles. **(A)** Sequence of wildtype *Wnk* (top) and *Wnk*^*AA*^ (bottom) with the RFSV motif in blue and the AA mutation in red. Area shaded in grey represents the introduced, silent NheI site. **(B, C)** Sequence traces of *Wnk*^*AA#16*^ (B) and *Wnk*^*AA#21*^ (C) alleles show the expected mutation of RF to AA (areas shaded in blue).
(TIF)

S3 Fig **(A)** Western blot of adult head lysates of *w*^*1118*^ control flies or flies expressing indicated HA-tagged Wnk constructs (upper panel: anti-HA blot; lower panel: blot reprobed for αTubulin as loading control). **(B)** Quantification of expression levels of Wnk deletion constructs normalized to the expression of HA-Wnk (biological triplicates).
(TIF)

S1 Table Detailed genotypes. (DOCX)

S2 Table Fly strains and plasmids. (XLSX)

S3 Table Oligonucleotides. (DOCX)
