# Supplementary figures and images for: Unanticipated domain requirements for Drosophila Wnk kinase in vivo
Source: PLoS Genet. 2023 Oct 11;19(10):e1010975. doi: 10.1371/journal.pgen.1010975 (PMC10593226; doi:10.1371/journal.pgen.1010975)

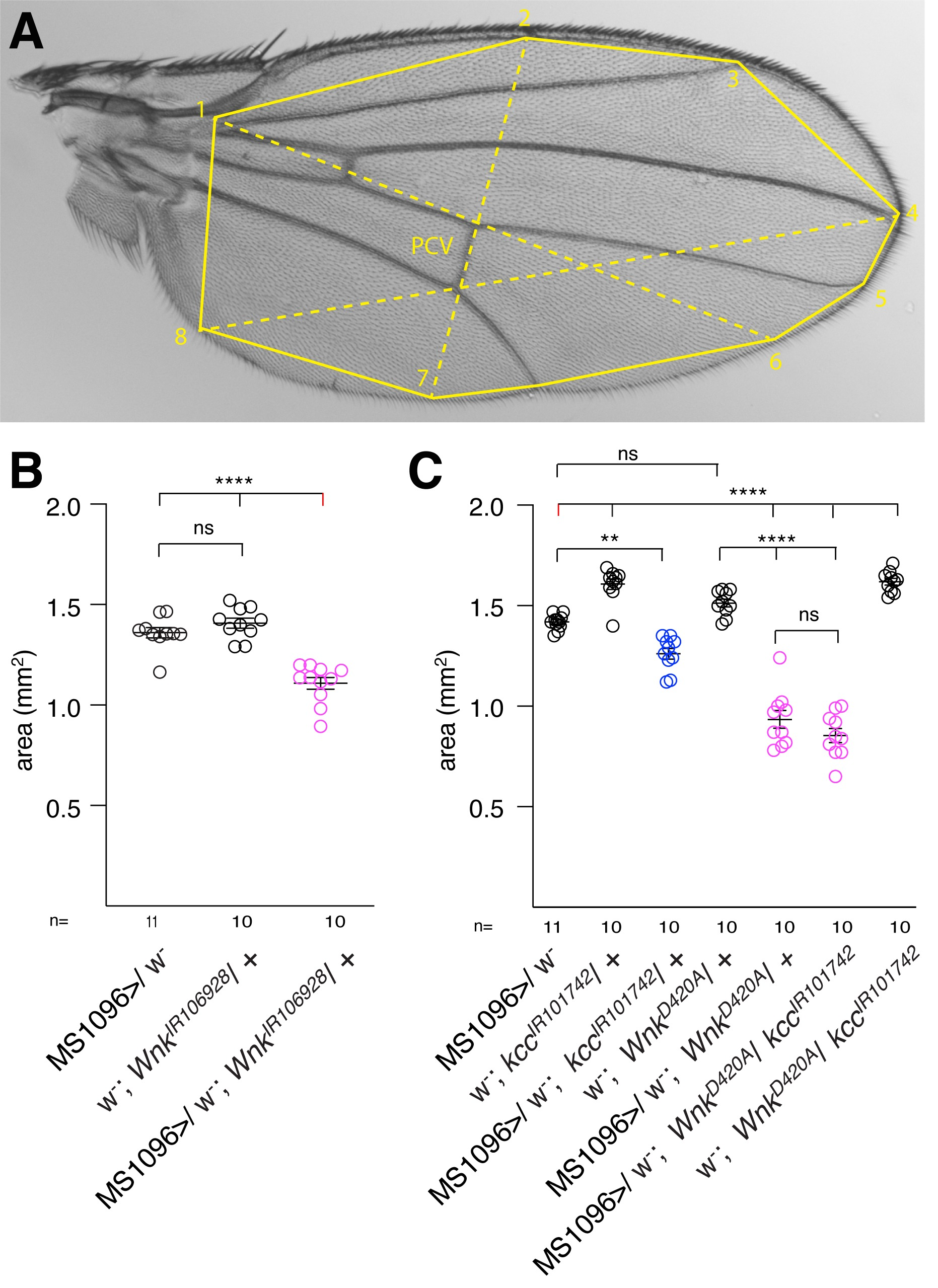

Supplement: S1 Fig — (A) Schematic outlining the wing area quantified using landmarks. #1, 3: beginning and end of wing vein L2; #4, 5 mark the ends of L3 and L4, respectively; #2, 7: wing margin crossing points of hypothetical extension of posterior cross vein (PCV); #6: margin crossing of a line extended from #1 to the beginning of the PCV on L4; #8: margin crossing of a line extended from #4 through the intersection of the PCV with L5. (B, C) Quantification of wing sizes of indicated genotypes. (B) Knockdown of Wnk in the whole wing pouch by MS1096-Gal4 causes a reduction of the total wing size (B), as does expression of dominant-negative WnkD420A (C). Compared to controls, knockdown of kcc in the wing pouch slightly reduces wing size on its own (C), but does not affect the size reduction due to WnkD420A. One-way ANOVA (Tukey correction) P <0.0001. ****, P <0.0001; **, P <0.01; ns, not significant. Only relevant comparisons are shown. (TIF) [file pgen.1010975.s001.tif]

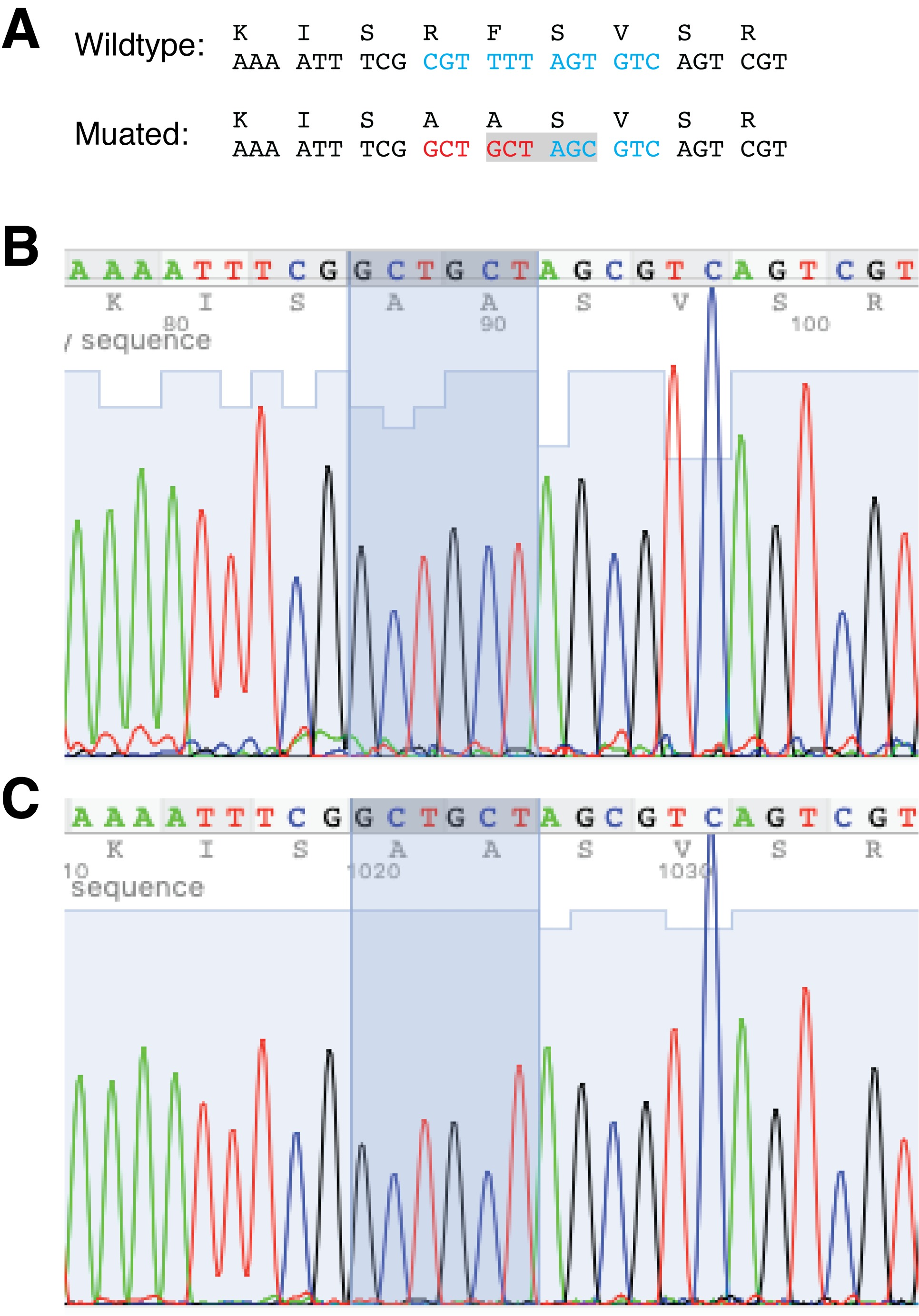

Supplement: S2 Fig — (A) Sequence of wildtype Wnk (top) and WnkAA (bottom) with the RFSV motif in blue and the AA mutation in red. Area shaded in grey represents the introduced, silent NheI site. (B, C) Sequence traces of WnkAA#16 (B) and WnkAA#21 (C) alleles show the expected mutation of RF to AA (areas shaded in blue). (TIF) [file pgen.1010975.s002.tif]

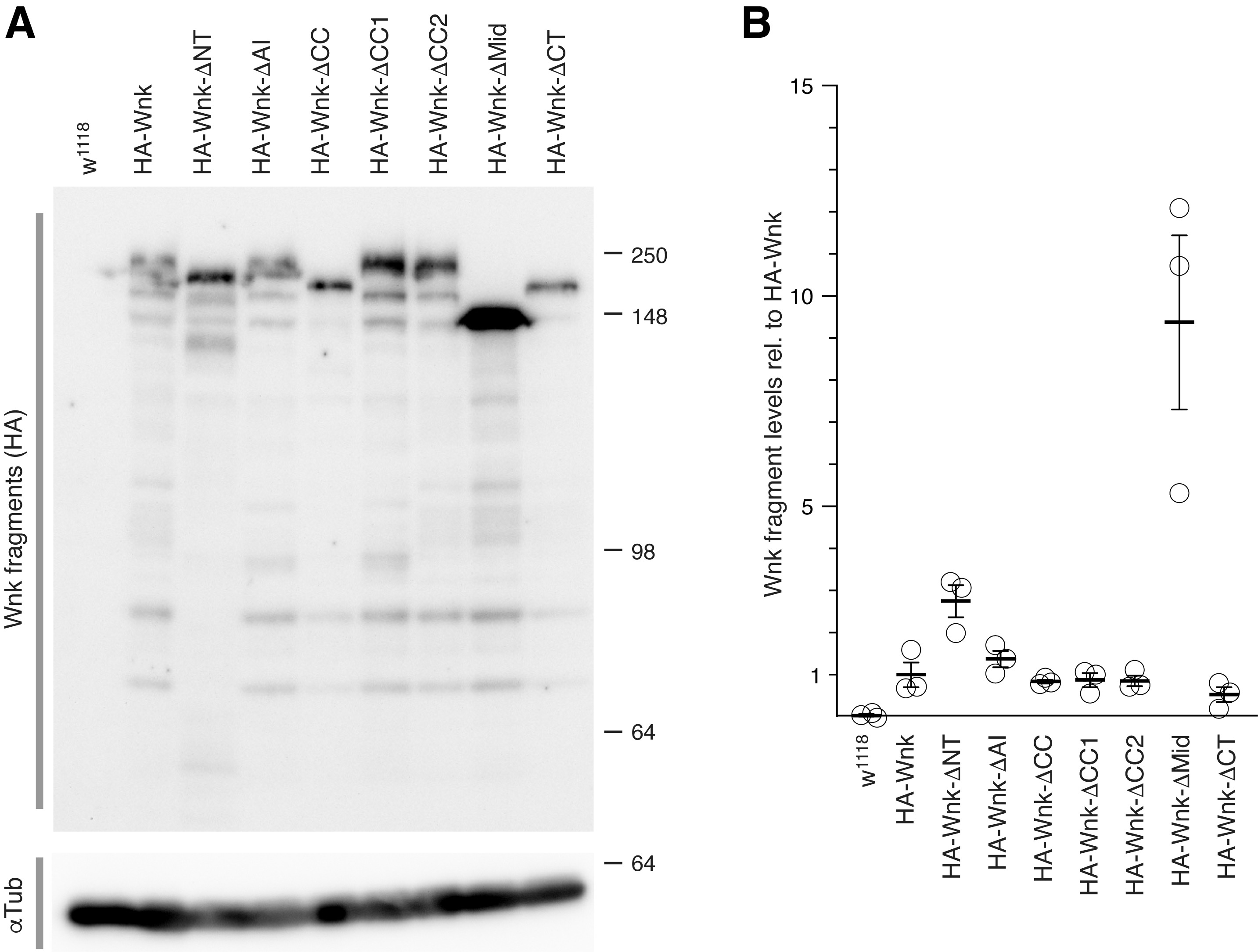

Supplement: S3 Fig — (A) Western blot of adult head lysates of w1118 control flies or flies expressing indicated HA-tagged Wnk constructs (upper panel: anti-HA blot; lower panel: blot reprobed for αTubulin as loading control). (B) Quantification of expression levels of Wnk deletion constructs normalized to the expression of HA-Wnk (biological triplicates). (TIF) [file pgen.1010975.s003.tif]
