# Supplementary material for: Unanticipated domain requirements for Drosophila Wnk kinase in vivo
Source: PLoS Genet. 2023 Oct 11;19(10):e1010975. doi: 10.1371/journal.pgen.1010975 (PMC10593226; doi:10.1371/journal.pgen.1010975)
Supplement: S1 Table — (DOCX) [file pgen.1010975.s004.docx]

**Supporting Table 1 Genotypes**

**Genotypes:**

**Figure 1**

**A:** *w^1118^; enGal4*

**B:** *w^1118^; enGal4 / attP^22A^ UAS-Fray^D185A^*

**C:** *w^1118^; enGal4, UAS-Wnk^IR106928^ / +*

**D:** *w^1118^; enGal4, UAS-Wnk^IR106928^* */ attP^22A^ UAS-Fray^T206E^*

**E:** *w^1118^; enGal4, UAS-Wnk^IR106928^* */ attP^22A^ UAS-Fray^D185A^*

**F:** *w^1118^; enGal4, UAS-Wnk^IR106928^  / attP^22A^ UAS-Fray*

**I:** *w^1118^; enGal4, UAS-Wnk^IR106928^* */ attP^22A^ UAS-hsWNK2*

**Figure 2**

**A:** *w^1118^; enGal4 /+*

**B:** *w^1118^; enGal4, UAS-Wnk^IR106928^ / +*

**C:** *w^1118^; enGal4, UAS-Wnk^IR106928^ / attP^22A^ UAS-Fray*

**D:** *w^1118^; enGal4, UAS-Wnk^IR106928^* */ attP^22A^ UAS-Fray^D185A^*

**E:** *w^1118^*

*w^1118^; enGal4 /+*

*w^1118^; enGal4, UAS-Wnk^IR106928^ / +*

*w^1118^; ; frayr2 / +*

*w^1118^; enGal4 /+; frayr2 / +*

*w^1118^; enGal4, UAS-Wnk^IR106928^ / +; frayr2 / +*

*w^1118^; ; Df (ED2)^fray^*

*w^1118^; enGal4 /+; Df (ED2)^fray^*

*w^1118^; enGal4, UAS-Wnk^IR106928^ / +; Df (ED2)^fray^*

*w^1118^; UAST-Fray*

*w^1118^; enGal4 / UAST-Fray*

*w^1118^; enGal4, UAS-Wnk^IR106928^ / UAST-Fray*

*w^1118^; UAST-Fray^D185A^*

*w^1118^; enGal4 / UAST-Fray^D185A^*

*w^1118^; enGal4, UAS-Wnk^IR106928^ / UAST-Fray^D185A^*

**F:** *Ncc69^r2^*/ *Ncc69^r2^*

**G:** *w^1118^; enGal4, UAS-Wnk^IR106928^ / +; Ncc69^r2^*/ *Ncc69^r2^*

**H:** *w^1118^*

*w^1118^; enGal4 /+*

*w^1118^; enGal4, UAS-Wnk^IR106928^ / +*

*w^1118^; enGal4 /+; Ncc69^r2^*/ *+*

*w^1118^; enGal4, UAS-Wnk^IR106928^ / +; Ncc69^r2^*/ *+*

*w^1118^; enGal4, UAS-Wnk^IR106928^ / +; Ncc69^r2^*/ *Ncc69^r2^*

*w^*^; ; Ncc69^r2^*/ *Ncc69^r2^*

**I:** *w^1118^*

*w^1118^; enGal4 / +*

*w*; Ncc69^r2^*/ *Ncc69^r2^*

*w^*^; enGal4 / +; Ncc69^r2^*/ *+*

**J:** *w^1118^; enGal4 / UAS-GFP*

**K:** *w^1118^; enGal4, UAS-Wnk^IR106928^ / UAS-GFP*

**L, M:** *w^1118^; enGal4 / UAS-GFP*

*w^1118^; enGal4, UAS-Wnk^IR106928^ / UAS-GFP*

**Figure 3**

**A:** *w^1118^*

**B:** *w^1118^; enGal4, UAS-Wnk^IR106928^ / +*

**C:** *w^1118^; enGal4, UAS-Wnk^IR106928^ / attP^22A^ UAS-DmWnk*

**D:** *w^1118^; enGal4, UAS-Wnk^IR106928^* */ attP^51D^ UAS-HsWNK2*

**E:** *w^1118^; enGal4, UAS-Wnk^IR106928^* */ attP^51D^ UAS-MmWnk4*

**F:** *w^1118^; enGal4, UAS-Wnk^IR106928^* */ attP^51D^ UAS-RnWnk1*

**Figure 4**

**A:** *w^1118^*

**B, C:** *w^1118^*

*w^1118^; enGal4, UAS-Wnk^IR106928^ / +*

*w^1118^; enGal4, UAS-Wnk^IR106928^ / attP^22A^ UAS-Fray*

*w^1118^; enGal4, UAS-Wnk^IR106928^* */ attP^22A^ UAS-Fray^D185A^*

*w^1118^; enGal4, attP^22A^ UAS-Fray^D185A^*

**Figure 5**

**B, C:** *w^-^; Df(3L)ED4978 / Df(3L)ED4978*

*w^-^; Df(3L)ED4978 / Wnk^ex22^ FRT80*

*w^-^; Df(3L)ED4978 / Wnk^MB06499^ FRT80*

*w^-^; Wnk^ex22^ FRT80 / Df(3L)ED4978*

*w^-^; Wnk^ex22^ FRT80 / Wnk^ex22^ FRT80*

*w^-^; Wnk^ex22^ FRT80 / Wnk^MB06499^ FRT80*

*w^-^; Wnk ^MB06499^ FRT80 / Df(3L)ED4978*

*w^-^; Wnk ^MB06499^ FRT80 / Wnk^ex22^ FRT80*

*w^-^; Wnk ^MB06499^ FRT80 / Wnk^MB06499^ FRT80*

*w^-^; Wnk^AA#16^ / Df(3L)ED4978*

*w^-^; Wnk ^AA#16^ / Wnk^ex22^ FRT80*

*w^-^; Wnk ^AA#16^ / Wnk^MB06499^ FRT80*

*w^-^; Wnk^AA#21^ / Df(3L)ED4978*

*w^-^; Wnk ^AA#21^ / Wnk^ex22^ FRT80*

*w^-^; Wnk ^AA#21^ / Wnk^MB06499^ FRT80*

*w^-^; Wnk^dsRed#16^ / Df(3L)ED4978*

*w^-^; Wnk ^dsRed#16^ / Wnk^ex22^ FRT80*

*w^-^; Wnk ^dsRed#16^ / Wnk^MB06499^ FRT80*

**D:** eyFLP /+; ; *Wnk^ex22^ FRT80 / P[w+] FRT80*

**E:** eyFLP /+; ; *Wnk^AA#16^ FRT80 / P[w+] FRT80*

**Figure 6**

**A, B:** *w^Berlin^; attP^40^ UAS-Wnk^IR42521^ attP^51D^ UAS-RnSPAK^D219A^/*+; *c42-Gal4/+*

*w^Berlin^; attP^51D^ UAS-RnSPAK^D219A^/+; c42-Gal4/+*

*w^Berlin^; attP^51D^ UAS-RnSPAK^D219A^/+; c42-GAL4, Wnk^MB06499^ / Wnk^AA#21^*

**C-H:** *w^Berlin^;*

*w^Berlin^; Wnk^MB06499^ / +*

*w^Berlin^; Wnk^AA#21^ / +*

*w^Berlin^; Wnk^MB06499^ / Wnk^AA#21^*

**Figure 7**

w^-^; *attP40 Tub-HA-Wnk* / +; *Wnk^dsRed#16^* / *Df(3L)ED4978*

w^-^; *attP40 Tub-HA-Wnk* / +; *Wnk^dsRed#16^* / *Wnk^ex22^ FRT80*

w^-^; *attP40 Tub-HA-Wnk* / +; *Wnk^dsRed#16^* / *Wnk^MB06499^ FRT80*

w^-^; *attP40 Tub-HA-Wnk* / +; *Wnk^ex22^ FRT80* / *Df(3L)ED4978*

w^-^; *attP40 Tub-HA-Wnk* / +; *Wnk^MB06499^ FRT80*/ *Df(3L)ED4978*

w^-^; *attP40 Tub-HA-ΔNT* / +; *Wnk^dsRed#16^* / *Df(3L)ED4978*

w^-^; *attP40 Tub-HA-ΔNT* / +; *Wnk^dsRed#16^* / *Wnk^ex22^ FRT80*

w^-^; *attP40 Tub-HA-ΔNT* / +; *Wnk^dsRed#16^* / *Wnk^MB06499^ FRT80*

w^-^; *attP40 Tub-HA-ΔNT* / +; *Wnk^ex22^ FRT80* / *Df(3L)ED4978*

w^-^; *attP40 Tub-HA-ΔNT* / +; *Wnk^MB06499^ FRT80*/ *Df(3L)ED4978*

w^-^; *attP40 Tub-HA-ΔAI* / +; *Wnk^dsRed#16^* / *Df(3L)ED4978*

w^-^; *attP40 Tub-HA-ΔAI* / +; *Wnk^dsRed#16^* / *Wnk^ex22^ FRT80*

w^-^; *attP40 Tub-HA-ΔAI* / +; *Wnk^dsRed#16^* / *Wnk^MB06499^ FRT80*

w^-^; *attP40 Tub-HA-ΔAI* / +; *Wnk^ex22^ FRT80* / *Df(3L)ED4978*

w^-^; *attP40 Tub-HA-ΔAI* / +; *Wnk^MB06499^ FRT80*/ *Df(3L)ED4978*

w^-^; *attP40 Tub-HA-ΔCC* / +; *Wnk^dsRed#16^* / *Df(3L)ED4978*

w^-^; *attP40 Tub-HA-ΔCC* / +; *Wnk^dsRed#16^* / *Wnk^ex22^ FRT80*

w^-^; *attP40 Tub-HA-ΔCC* / +; *Wnk^dsRed#16^* / *Wnk^MB06499^ FRT80*

w^-^; *attP40 Tub-HA-ΔCC* / +; *Wnk^ex22^ FRT80* / *Df(3L)ED4978*

w^-^; *attP40 Tub-HA-ΔCC* / +; *Wnk^MB06499^ FRT80*/ *Df(3L)ED4978*

w^-^; *attP40 Tub-HA-ΔCC1* / +; *Wnk^dsRed#16^* / *Df(3L)ED4978*

w^-^; *attP40 Tub-HA-ΔCC1* / +; *Wnk^dsRed#16^* / *Wnk^ex22^ FRT80*

w^-^; *attP40 Tub-HA-ΔCC1* / +; *Wnk^dsRed#16^* / *Wnk^MB06499^ FRT80*

w^-^; *attP40 Tub-HA-ΔCC1* / +; *Wnk^ex22^ FRT80* / *Df(3L)ED4978*

w^-^; *attP40 Tub-HA-ΔCC1* / +; *Wnk^MB06499^ FRT80*/ *Df(3L)ED4978*

w^-^; *attP40 Tub-HA-ΔCC2* / +; *Wnk^dsRed#16^* / *Df(3L)ED4978*

w^-^; *attP40 Tub-HA-ΔCC2* / +; *Wnk^dsRed#16^* / *Wnk^ex22^ FRT80*

w^-^; *attP40 Tub-HA-ΔCC2* / +; *Wnk^dsRed#16^* / *Wnk^MB06499^ FRT80*

w^-^; *attP40 Tub-HA-ΔCC2* / +; *Wnk^ex22^ FRT80* / *Df(3L)ED4978*

w^-^; *attP40 Tub-HA-ΔCC2* / +; *Wnk^MB06499^ FRT80*/ *Df(3L)ED4978*

w^-^; *attP40 Tub-HA-ΔMid* / +; *Wnk^dsRed#16^* / *Df(3L)ED4978*

w^-^; *attP40 Tub-HA-ΔMid* / +; *Wnk^dsRed#16^* / *Wnk^ex22^ FRT80*

w^-^; *attP40 Tub-HA-ΔMid* / +; *Wnk^dsRed#16^* / *Wnk^MB06499^ FRT80*

w^-^; *attP40 Tub-HA-ΔMid* / +; *Wnk^ex22^ FRT80* / *Df(3L)ED4978*

w^-^; *attP40 Tub-HA-ΔMid* / +; *Wnk^MB06499^ FRT80*/ *Df(3L)ED4978*

w^-^; *attP40 Tub-HA-ΔCT* / +; *Wnk^dsRed#16^* / *Df(3L)ED4978*

w^-^; *attP40 Tub-HA-ΔCT* / +; *Wnk^dsRed#16^* / *Wnk^ex22^ FRT80*

w^-^; *attP40 Tub-HA-ΔCT* / +; *Wnk^dsRed#16^* / *Wnk^MB06499^ FRT80*

w^-^; *attP40 Tub-HA-ΔCT* / +; *Wnk^ex22^ FRT80* / *Df(3L)ED4978*

w^-^; *attP40 Tub-HA-ΔCT* / +; *Wnk^MB06499^ FRT80*/ *Df(3L)ED4978*

**Figure S1**

**A:** *MS1096>/ w^Berlin^*

*w^Berlin^; Wnk^IR106928^/ +*

*MS1096>/ w^Berlin^; Wnk^IR106928^/ +*

**B:** *MS1096>/ w^Berlin^*

*w^Berlin^; kcc^IR101742^/ +*

*MS1096>/ w^Berlin^; kcc^IR101742^/ +*

*w^Berlin^; Wnk^D420A^/ +*

*MS1096>/ w^Berlin^; Wnk^D420A^/ +*

*MS1096>/ w^Berlin^; Wnk^D420A^/ kcc^IR101742^*

*w^Berlin^; kcc^IR101742^/ +*

**Figure S3**

w^-^; CyO/ +

w^-^; *attP40 Tub-HA-Wnk* / *+*

w^-^; *attP40 Tub-HA-ΔNT* / *+*

w^-^; *attP40 Tub-HA-ΔAI* / *+*

w^-^; *attP40 Tub-HA-ΔCC* / *+*

w^-^; *attP40 Tub-HA-ΔCC1* / *+*

w^-^; *attP40 Tub-HA-ΔCC2* / *+*

w^-^; *attP40 Tub-HA-ΔMid* / *+*

w^-^; *attP40 Tub-HA-ΔCT* / *+*
