# Supplementary material for: Unanticipated domain requirements for Drosophila Wnk kinase in vivo
Source: PLoS Genet. 2023 Oct 11;19(10):e1010975. doi: 10.1371/journal.pgen.1010975 (PMC10593226; doi:10.1371/journal.pgen.1010975)
Supplement: S3 Table — (DOCX) [file pgen.1010975.s006.docx]

**Supporting Table 3 Oligonucleotides**

Wnk deletion constructs

Wnk-RO-Fw-NotI-HA: ATAAGAATGCGGCCGCCACCATGTACCCCTACGACGTGCCCGACTACGCGATGGATAGCAAAATAGAACCTGCAG

Wnk-RO-Rv-Avr: AGATTCCTAGGTTATGTGTTTGCGGAGCTAGTG

WnkRO-Nterm-Del-Fw: GACGATGATCCGGTGGCCATGTCG

WnkRO-Nterm-Del-Fw: CACCGGATCATCGTCCATCGCGTAGTCGGGCACGTCGTA

WnkRO-StuI-Fw: TGTCGCCAGATACTAAAAGGCCTTAACTTCCTGCATACTC

WnkRO-XbaI-Rv: TTCTATTTTTAACTGCTCTAGAGTGTTGGCCGCTCCAGTTC

WnkRO-Del-AI-Fw: GTGCAGGTGGTGTCGTTGCTTA

WnkRO-Del-AI-Rv: TGATGTTGTTGCTGAGGCATAAGCAACGACACCACCTGCA

WnkRO-CCdelete-Fw: ATGCCTCAGCAACAACATCA

WnkRO-CCdelete-Rv: TGATGTTGTTGCTGAGGCATAAGCAACGACACCACCTGCA

WnkCC1del-Fw: AGTTTGCCGACTAACGTCGATGAA

WnkCC1del-Rv: GTTAGTCGGCAAACTAAGCAACGACACCACCTGCACTTT

WnkCC2NewDel-Fw: ATGCCTCAGCAACAACATCAGCAA

WnkCC2NewDel-Rv: TTGTTGCTGAGGCATCAGTTGTTGCTGCATTATCTGTTG

WnkRO-mid-del-Fw: CCTCCGCAAAGGAATCCCAGC

WnkRO-mid-del-Rv: GCTGGGATTCCTTTGCGGAGGAACGAGTTGCTGATGTTGTT

WnkRO_NdeI_Rv: GGCACGTTTTGTTGGTGCATATGCGACTGCATCTGCAACTG

WnkRO-StopAvrII-Fw: ATGATAACCTAGGTGG

WnkRO-StopAvrII-Rv: CCTAGGTTATCATCCA

Expression constructs

pENTR3CWnkRO_Fw: TGTACAAAAAAGCAGGCTCTATGGATAGCAAAATAGAACCTGCA

pENTR3CWnkORF_N_midR: GCTGGGTCTAGATATCTCGAGTCGACGCTGGTTACACTAAG

pENTR3CWnkORF_C_midFW: TGTACAAAAAAGCAGGCTCTGTCGACGAGGGCAGCGTCAT

pENTR3CWnkRO_Rv: TGTACAAAAAAGCAGGCTCTATGGATAGCAAAATAGAACCTGCA

wnk_RFtoAA_FW: CGCGCGTAAAATTTCGGCTGCTAGCGTCAGTCGTGTGCAGGAGCAGAAAACTTCAACCGGAGTTGAGGAACCAGCTCAAG

GTCAACTTAAGATCGACCTCCAAGTCGCCGGCCCTGG

wnk_RFtoAA_RW: GGGCCGGCGACTTGGAGGTCGATCTTAAGTTGACCTTGAGCTGGTTCCTCAACTCCGGTTGAAGTTTTCTGCTCCTGCAC

ACGACTGACGCTAGCAGCCGAAATTTTACG

Wnk RF(x)V in vivo targeting

RFxV-leftArm_for_hom: CTATAGTGTCTTCGGGGCCGATTGGACACTTCGGAAAACG

RFxV-leftArm_mut_rev: GATTATCTTTCTAGGGTTAAGTTGACCTTGAGCTGGTTCCTCAACTCCGGTTGAAGTTTTCTGCTCTTGCACACGACTGA

CGCTAGCAGCCGAAATTTTACGCGCGCTTG

RFxV-RightArm_for_hom: CGCAGACTATCTTTCTAGGGTTAAGATCGACCTCCAAGTCGCCGG

RFxV-rightArm_rev_hom: ATATGGTCTTCTTTTCCCGGAGGCTCTTCGGTAACTGCAA

pCFD3_wnk_gRNA_sense: GTCGTTTTAGTGTCAGTCGTGTGC

pCFD3_wnk_gRNA_anti: AAACGCACACGACTGACACTAAAA

Wnk-cr_seq-Left-arm-Fw: AATTTGCCCTTGAATCGTCA

Wnk_cr_seq-Right-arm-Rv: CGCGTATTGCACAATTGGTT
